# In the long shadow of our best intentions: Model-based assessment of the consequences of school reopening during the COVID-19 pandemic

**DOI:** 10.1371/journal.pone.0248509

**Published:** 2021-03-25

**Authors:** Kaitlyn E. Johnson, Madison Stoddard, Ryan P. Nolan, Douglas E. White, Natasha S. Hochberg, Arijit Chakravarty

**Affiliations:** 1 Department of Integrative Biology, The University of Texas at Austin, Austin, TX, United States of America; 2 Fractal Therapeutics, Cambridge, MA, United States of America; 3 Halozyme Therapeutics, San Diego, CA, United States of America; 4 Independent Researcher, Atlanta, GA, United States of America; 5 Department of Epidemiology, Boston University School of Public Health, Boston, MA, United States of America; 6 Department of Medicine, Boston University School of Medicine, Boston, MA, United States of America; University of Surrey, School of Veterinary Medicine, UNITED KINGDOM

## Abstract

As the world grapples with the ongoing COVID-19 pandemic, a particularly thorny set of questions surrounds the reopening of primary and secondary (K-12) schools. The benefits of in-person learning are numerous, in terms of education quality, mental health, emotional well-being, equity and access to food and shelter. Early reports suggested that children might have reduced susceptibility to COVID-19, and children have been shown to experience fewer complications than older adults. Over the past few months, our understanding of COVID-19 has been further shaped by emerging data, and it is now understood that children are as susceptible to infection as adults and have a similar viral load during infection, even if asymptomatic. Based on this updated understanding of the disease, we have used epidemiological modeling to explore the feasibility and consequences of school reopening in the face of differing rates of COVID-19 prevalence and transmission. We focused our analysis on the United States, but the results are applicable to other countries as well. We demonstrate the potential for a large discrepancy between detected cases and true infections in schools due to the combination of high asymptomatic rates in children coupled with delays in seeking testing and receiving results from diagnostic tests. Our findings indicate that, regardless of the initial prevalence of the disease, and in the absence of robust surveillance testing and contact-tracing, most schools in the United States can expect to remain open for 20–60 days without the emergence of sizeable disease clusters. At this point, even if schools choose to close after outbreaks occur, COVID-19 cases will be seeded from these school clusters and amplified into the community. Thus, our findings suggest that the debate between the risks to student safety and benefits of in-person learning frames a false dual choice. Reopening schools without surveillance testing and contact tracing measures in place will lead to spread within the schools and within the communities that eventually forces a return to remote learning and leaves a trail of infection in its wake.

## Introduction

As is to be expected with any emerging infectious disease, our understanding of the biology and transmission of COVID-19 continues to evolve rapidly during this ongoing pandemic. In particular, changes in our understanding of the disease impact our expectations of the risk to children and the community that would arise from the reopening of K-12 (primary and secondary) schools and colleges.

A number of studies at the outset of the pandemic suggested that children were less susceptible, with a lower risk of being infected with COVID-19 upon exposure to the virus [1]. Children and young adults in general were also found to have mild symptoms of the disease, with low rates of hospitalization and death [2–4]. For this demographic group, asymptomatic and pauci-symptomatic cases were also observed at a high frequency [3, 5–8]. Some early reports also suggested a lower rate of infection (attack rate) in children [9–11]. However, these findings were confounded with widespread school closures in the Spring of 2020 (Jing *et al*., 2020) and the potential for a bias in case detection due to undercounting of asymptomatic cases. These findings were also contradicted by other reports suggesting no difference in attack rates between children and adults [12]. A key finding reported and cited often in the early debate about school reopening was that children were not usually the index case (first infection) within in a family [13], suggesting that children may not be responsible for disease spread [1, 14].

Based on the scientific understanding at the time, and mindful of the harm to children’s long term development in the face of prolonged school closures, a number of medical associations and public health figures strongly advocated for a return to in-person schooling [15–18] even going so far as to endorse a return to in-person schooling with closer spacing than recommended by the CDC [16]. An in-depth white paper on the CDC’s own website also argues this point, emphasizing the harm to children that results from loss of in-person educational instruction and school resources [19]. The harm to children’s development, to their psychological well-being (particularly for teenagers), the potential risks to vulnerable children, and the increase in inequality that results from school closure is well documented [20–22] and frames a strong case for a return to in-person schooling if the biology of COVID-19 supports it.

In recent months, our fundamental understanding of the disease has shifted under our feet. First, a number of studies demonstrated that children’s susceptibility to COVID-19 is similar to that of adults [23–25], or only slightly lower [26], and there have been numerous publicized examples of peer-to-peer spread among children in congregate settings [27, 28], and multiple documented examples of secondary transmission in the in-school setting, both to and from children [29–32]. These findings suggest that the low attack rate observed in children during the early days of the pandemic may have been a function of school closures and other behavioral changes [10] rather than reduced susceptibility among children. Second, viral loads in children have been found to be similar or arguably higher than those of severely ill adults [33–35]. Third, and most tellingly, a large proportion of COVID-19 cases in children and young adults has been found to be asymptomatic [23, 36, 37]. This last finding casts further doubt on the early reports of lower attack rates and transmission from children, as asymptomatic cases were often missed in epidemiological tracing studies during the early stages of the pandemic.

Other aspects of our understanding of COVID-19 spread have also evolved over time. In the early days of the pandemic, guidelines for preventing the spread of the disease were heavily focused on respiratory droplets and transmission from fomites (objects contaminated with the virus). As our understanding of the disease has matured, fomites have been recognized to be less of a threat [38, 39]. On the other hand, airborne transmission via small aerosolized droplets has been identified as a plausible route of disease spread [40, 41]. There are multiple documented cases of indoor spread that can best be explained by airborne transmission (for a summary, see [42], and S4 Table in S1 File). First-principles calculations of viral load and droplet physics add further credence to the view that transmission via small aerosolized droplets represents a tangible threat in indoor environments [43], a view that is shared by the WHO [39] and CDC [44]. Consistent with this, COVID-19 is difficult to control in indoor settings, and spread can occur over short time periods, even in the presence of extreme precautions [45]. One report based on contact tracing of clusters of cases occurring in Japan estimated the odds of transmission in an enclosed environment to be 18.7 fold higher than in an outdoor environment [46], at a time (February 2020) when mask-wearing in Japan may have been generally prevalent [47].

While our understanding of SARS-CoV-2 transmission has altered rapidly over the past few months, guidance for infection prevention has not kept pace. The CDC now acknowledges that the disease can be spread in poorly ventilated indoor spaces [44], even at distances greater than six feet. However, the guidance continues to emphasize the six-foot rule, which was originally intended to minimize exposure to cough and sneeze trajectories. Thus the guidance is not necessarily adequate to limit SARS-CoV-2 spread [42]. Guidance also strongly emphasize handwashing and surface cleaning, even though the CDC’s recent statements have emphasized that surface contamination is not a main driver of SARS-CoV-2 spread [44]. Many schools are still following the original CDC guidelines from the early days of the disease [48, 49]. To the extent that a gap has now opened up between the CDC’s understanding of the disease [44] and the official guidelines for school reopening [49], the effectiveness of these guidelines- even for schools with the resources to follow them perfectly- is unknown.

Taken together, this state of affairs raises the possibility that children may be a potential source of contagion for COVID-19, and schools -despite following guidelines- may be inadequately prepared for this threat. An outbreak seeded among children may in fact result in transmission chains that are harder to bring under control, as the asymptomatic nature and milder presentation of infected children will delay the detection of the disease in the absence of widespread, rapid, molecular testing.

With this in mind, we have conducted a model-based investigation of the feasibility and consequences of school reopening within the United States during the SARS-CoV-2 pandemic. We used a Susceptible-Exposed-Infectious-Recovered (SEIR) epidemiological model to examine the potential consequences of opening schools at various school reproductive numbers and infection prevalence. We evaluate the magnitude of the potential discrepancy between detected cases and true infections, and assess the consequences of seeding these additional infections in the community.

## Methods

### SEIR model of SARS-CoV-2 within schools

Our analysis was based on a standard susceptible-exposed-infected recovered (SEIR) epidemiological model [50, 51]. We assumed an equivalent level of infectivity and susceptibility between children and adults, a conservative assumption in the face of our updated understanding of the situation in children for the current pandemic. Susceptible individuals (S) can become infected at a rate proportional to the number of infected individuals. We assumed a constant infectiousness throughout the duration of infection, as well as a constant rate of recovery and progression from latency to infectiousness, thus implying exponential latent and infectious periods. Although our model represents a simplified approximation for SARS-CoV-2 dynamics [52–54], we anticipate that these simplifying assumptions are not likely to have a major effect on the outcomes of interest which are population-level outbreak kinetics in the absence of intervention at specific times. We assumed that robust contact-tracing [55] and routine surveillance testing [56] were not present in school settings to focus on this specific common scenario. Susceptible individuals (S) become exposed (E) at a transmission rate (β) proportional to the number of individuals infected (I). Infected individuals were assumed to remain exposed but not detectable or infectious (E) for an average of 3 days, corresponding to a rate α [53]. A proportion of the infected individuals (I) develop symptoms after an average of 2.3 days of being infectious [54]. Infectious individuals recover after an average of 14 days, corresponding to a rate of γ [54]. The model equations are as follows:

$$\frac{dS}{dt}=-\frac{\beta SI}{N}$$

$$\frac{dE}{dt}=\frac{\beta SI}{N}-\alpha E$$

$$\frac{dI}{dt}=\alpha E-\gamma I$$

$$\frac{dR}{dt}=\gamma I$$

Where *N* = total size of the school population (See S1 Table in S1 File for description of model parameters). The model assumes that the students are a well-mixed population, and therefore does not take into account any cohorting or podding. The model does not explicitly account for reduced transmission on the weekends, and so all references to days corresponds to total epidemic days, not school days. The initial number of infected individuals in a school is set as equivalent to the community prevalence. We did not explicitly account for stochastic introductions into the school population during the outbreak, instead assuming that outbreak dynamics were mainly driven by initial conditions, consistent with recent studies that show minimal impact of international travel restrictions once an epidemic has already been significantly seeded in a community [57, 58].

### Estimation of time to school closure

To simulate a school closing, we had to make some assumptions about the “tolerance” of a school district to detected COVID-19 cases. Based on recent university closings [59–61], we reasoned that a cumulative incidence of detected cases exceeding 1% of the student body would initiate moving schools online. This corresponds to 10 students in a school of 1000. We reasoned that each student had on average 10 contacts who would be told to quarantine for 14 days upon case detection. We note that this does not account explicitly for any podding or cohorting measures in place that may violate the well-mixed population assumption and prevent the need to close all sections of the school population. We report the true number of cumulative student infections at the time that the school closes.

### Estimation of detected cases in the school and community

To simulate the expected number of detected cases over time, we transformed the infections over time in the school and community from the infected compartment of the SEIR model in the following ways. First, we assumed cases are only detected via symptomatic individuals seeking testing after symptom onset. Proactive surveillance testing at schools was not assumed, as this is not part of CDC guidelines for school reopening [49], and many public K-12 schools in the country were not performing surveillance testing at the time of this writing. Based on age-structured estimates of symptomatic rates, we assumed students develop symptoms in only 21% of cases [62]. In the infectious individuals that eventually develop symptoms, the time to detect cases is delayed. We assumed a 2.3 day delay from start of infectiousness to symptom onset, and a further optimistic 0.7 day delay until seeking a test. Then we assumed the delay to receive test results was an additional 4 days, consistent with the national average as of August 2020 [63]. We make the simplifying assumption that after 3 days of infectiousness before seeking a test and 4 days of a delay for test results, isolation of positives is unlikely to have a significant effect on reducing transmission [64, 65]. We do not explicitly take into account contact-tracing systems within schools and communities to further reduce transmission, although we acknowledge the significant role that contact-tracing could play in reducing transmission of SARS-CoV-2 [66]. However, given conditions at the time of this writing (January 2021), we assumed few contact-tracing programs were present at the scale needed to significantly reduce transmission [56].

Using the partial reporting based on symptomatic rates and the delay to seek and receive test results, we report the detected infections alongside the true infection prevalence. For each initial prevalence and school reproductive number, we report the estimated true infections at the time that the first case is detected (S2 Fig in S1 File).

### Estimation of average initial prevalence in schools

The average initial prevalence of infection in students is assumed to be equal to that of their community. The number of expected initial infections was calculated from reported county cases following the method originally described by researchers at the University of Texas at Austin [67, 68]. To calculate the put the prevalence scenarios explored in context to US counties, we used the New York Times database of daily cases per 100k by county. We assumed an infectious duration of 7 days and a reporting rate of 1 in 5 (with scenario lower and upper bounds of 1 in 3 and 1 in 10). We assumed the initial students that would arrive infected in the first week in a school of 1000 would be sampled evenly from this county prevalence [69]. As a specific motivating example, we considered the scenario that was present in the US in September 2020. A table of county prevalence values for some representative regions in the US are shown in S2 Table in S1 File, and an example of how to estimate these from the New York Times daily case counts per 100k in each county is demonstrated. For simulations varying the effect of R_0_ on infections, we assumed a baseline prevalence close to the national prevalence of 5 in 1000 as of August 27th, 2020 [70] with upper and lower scenario bounds of 10 in 1000 and 3 in 1000 respectively.

### Estimation of school R_0_

It is not well-known empirically what a school’s basic reproductive number (R_0_), the number of secondary infections from a single infected student, is at baseline due to the lack of early epidemic data. We reasoned that schools would vary significantly in their ability to mitigate transmission, partially as a function of their resources. As of September 2020, there had been multiple documented cases of schools across the country reopening without providing adequate personal protective equipment (PPE), mandating mask use or enforcing physical distancing [71–73]. On the other hand, some schools have gone beyond the CDC guidance, implementing hybrid schooling, upgrading their ventilation systems, mandating masks and providing adequate PPE to their students and staff. Modeling the impact of specific interventions on the R_0_ is beyond the scope of this paper, and we have instead varied the R_0_ over a wide range to encompass the range of mitigation measures in place, focusing our attention on the downstream consequences of reopening given a particular R_0_.

We reasoned that an indoor setting, where masks and distancing might vary in adherence, could have a range of R_0_ values from as low as just above 1 to as high as 5. (Some estimates of the R_0_ of SARS-CoV-2 in different settings are presented in S3 Table in S1 File for comparison, and some examples of evidence supporting efficient indoor transmission are provided in S4 Table in S1 File). For simulations varying the initial prevalence, we assumed a baseline R_0_ of 2.5, with upper and lower scenario bounds of 3.5 and 2.2 respectively.

Unfortunately, it is unlikely that schools will have any knowledge of their R_0_ prior to reopening. Thus, it is our suggestion that the reader considers the outcomes at both the low and the high end of the R_0_ range as being within the realm of the possible for their own situation.

### Estimation of effect of secondary spread within the community

Because student infections pose a significant risk of infecting their family members and thus the surrounding community, we performed an analysis to assess what this risk might be in terms of secondary community infections. Since school outbreaks are expected to occur fairly quickly, we treated the number of infections at school closure as the initial condition for a community epidemic in a community of 50,000 individuals, and used the same SEIR model structure to estimate the scale of these secondary cluster. For a given community R_0_ and number of initially seeded student infections, we then modeled the number of cumulative additional infections expected within the community in 100 days after the infections were seeded by the school. The presence of immune individuals within the community is implicitly incorporated into the low community R_0_, which may be due to a combination of immunity and social distancing measures.

## Results

### Clusters will develop quickly in schools upon reopening

**Fig 1:**
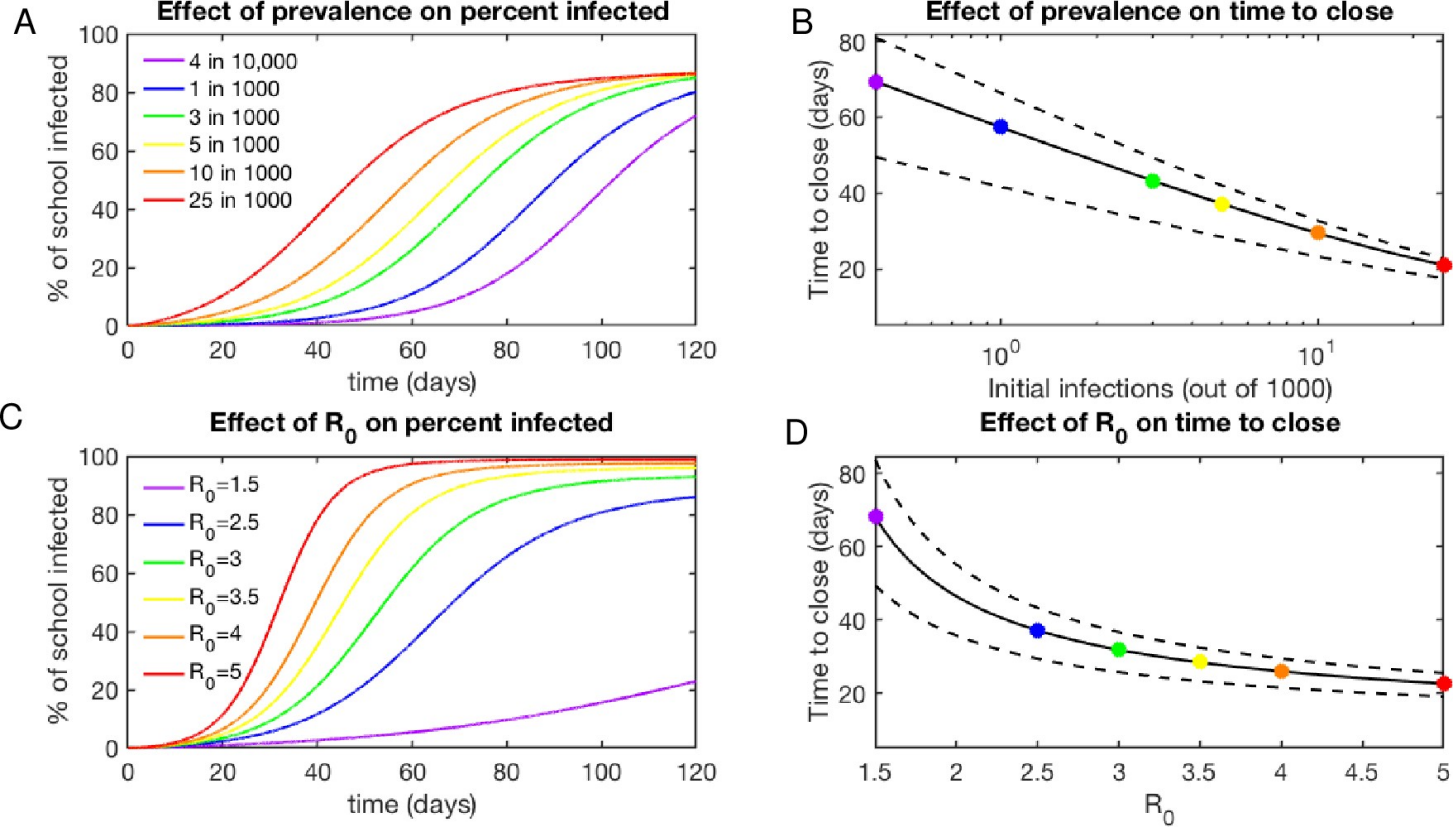
Clusters will develop quickly in schools upon reopening. A. Simulated time course of percent of school infected for initial confirmed prevalences ranging from 4 in 10,000 to 25 in 1000, demonstrating that a school reopening at low but non-zero prevalence of disease is simply delayed in its epidemic compared to schools with a higher initial disease prevalence. R_0_ = 2.5 for all projections. B. The effect of initial disease prevalence on the expected time to close (assuming a 1% threshold rate of detected cases is sufficient to trigger school closure), indicating that most schools in this regime will close between 20 and 70 days after opening. Upper and lower bounds reflect R_0_ bounds of 3.5 and 2.2 (See Methods: Estimation of school R_0_). C. Simulated time course of percent of school infected for reproductive numbers ranging from 1.5 to 5, demonstrating the speed at which high R_0_ s can lead to widespread infection. Initial prevalence is 5 in 1000 for all projections. D. The effect of R_0_ on the expected time to close, indicating again that most schools in this transmission regime will close between 20 and 70 days after opening. Upper and lower bounds reflect prevalence of 3 in 1000 and 10 in 1000 (See Methods: Estimation of average prevalence).

Our analysis suggests that most schools in the United States will experience outbreaks within a short time of reopening (Fig 1) for a wide range of initial disease prevalences and school R_0_. Focusing on Fig 1A and 1B, where school R_0_ was held constant at 2.5, we draw the reader’s attention to the line representing a prevalence of 1 in 1000 (blue line). This prevalence, which a number of localities have set as their “green zone” guideline for lowest concern (for example [74]), only results in a delay of 60 epidemic days until the outbreak reaches a state where more than 1% of the student body has detectable infections. Throughout this work, we made the assumption that a detectable outbreak of this size would prompt school closing due to large numbers of students and staff needing to be isolated or quarantined. In fact, some schools did close when cases were detected (the Boston and New York schools were among them [75, 76]), while others did not. Somewhat obviously, if a school chooses to remain open in the face of continued spread of the virus (without implementing aggressive measures to control spread, such as contact-tracing based isolation), it can expect about 60–80% of the students to eventually become infected regardless of initial prevalence. As an analytical example, the initial prevalence of disease explored were based on the range of initial prevalences observed in the US prior to school reopenings as of September 2020. It is worth noting that the national average prevalence of active infections of COVID-19 within the United States as of September 2020 was 5/1000, which corresponds to the yellow line in Fig 1A. At this prevalence, schools can expect to remain open for about 40 days with fewer than 1% of students with detected infections.

To determine the effect of transmission within the school on the rate of cluster growth and downstream consequences, we also examined the kinetics of cluster growth while varying the R_0_ across a reasonable range (R_0_ = 1–5) to be represent the breadth of possible mitigation measures in each school (Fig 1C). In this graph, the blue line marks an R_0_ of 2.5, a reasonable estimate for schools that have some measures in place to limit transmission. Schools reopening with an R_0_ = 2.5 can expect to close after 40 days on average, and sooner if their threshold for detected cases is lower or if staff illnesses and high quarantine rates force earlier closure. Across a wide range of conditions, the time to closing was 60 days or less (Fig 1D). Higher rates of transmission are certainly plausible for this disease in close quarters and will result in dramatically worse outcomes. The joint effect of varying school reproductive numbers and initial infection prevalence is demonstrated in S3 Fig in S1 File, demonstrating that only schools with an R_0_ below 1.5 and infection prevalence below 1 in 1000 can expect to remain open without large disease clusters for more than 100 days.

### Detected cases will form the tip of the iceberg

**Fig 2:**
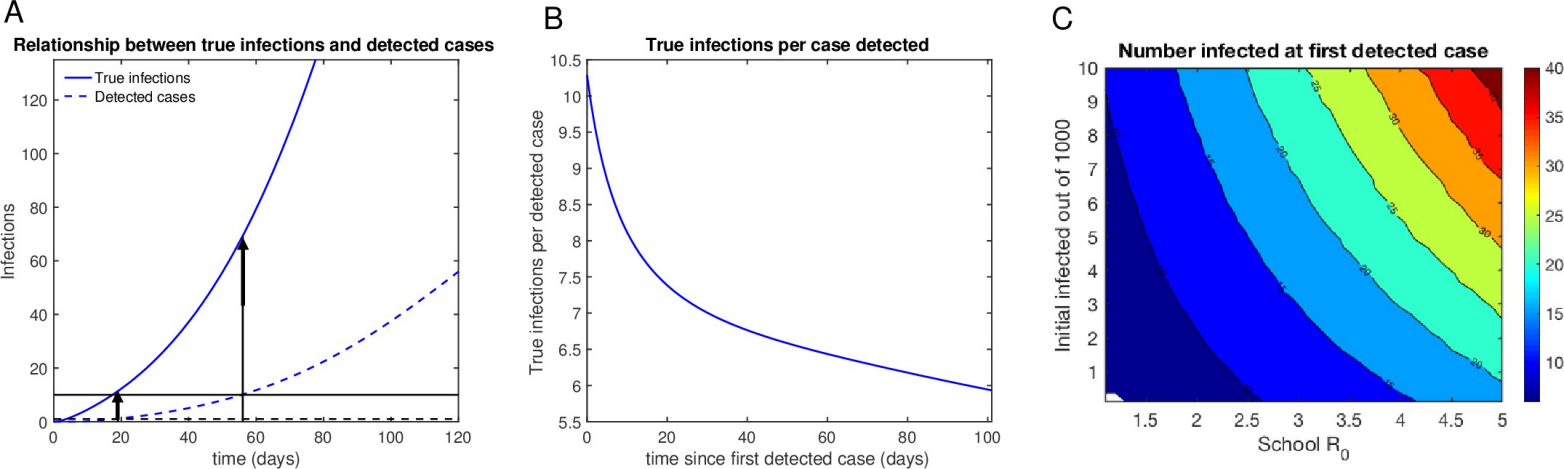
Detected cases will form the tip of the iceberg. A. Example of time course of infections (blue solid line) compared to time course of detected cases (blue dashed line), demonstrating a significant delay and under counting of reported student cases. Horizontal dashed black line represents the threshold for first detected case, and the horizontal black solid line represents the threshold for time to close of 10 detected cases in a school of 1000. Vertical arrows indicate the points on the true infection curve corresponding to the time of detection of the first case and school closure. In this example, at the time of the first detected case, more than 10 individuals are already infected, and at the time of detected 10 cases prompting school closure, more than 80 individuals are already infected. B. Number of true infections per case detected over time, indicating that early in the outbreak due to significant delays in being tested or receiving a diagnosis, true infections can be more than 10x greater than detected cases. C. Number infected out of 1000 at first detected case for different school R_0_s and infection prevalences, demonstrating the potential for a major discrepancy between true infections and detected cases at the time of first case detection.

Despite the formation of large disease clusters, in schools where systematic surveillance testing is not taking place, cases may be expected to mount undetected. Our modeling suggests that when clusters form in schools, the substantial contribution of asymptomatic and presymptomatic cases to transmission in the pediatric setting will create a discordance between the number of detected cases and the true scope of the outbreak (Fig 2A). This example demonstrates this discordance in a school with an initial disease prevalence of 1 in 1000 and a school R_0_ = 2.5.

This discordance is greatest at the beginning of the outbreak (Fig 2B), resulting in a 5 to 13-fold larger number of infections than detected cases at any time during the outbreak. As this ratio is highest at the beginning of the outbreak, it is precisely those schools that set the lowest thresholds (for the number of cases triggering closure) that will have the least understanding of the true scope of the outbreak at the time of closure. The number of true infections at the time of first detected case is shown as a function of school R_0_ and initial infection prevalence in Fig 2C and S2 Fig in S1 File. These finding has implications for controlling the secondary spread of COVID-19 through the community, as schools that shut down with only a few detected cases may still be seeding their communities with a larger number of infections than is appreciated at the time of school closure.

### Schools reopenings and eventual closures can seed community infections

To provide more context for the outcomes that can be expected, we explored a few case studies of simulated K-12 schools, each with 1000 students (Table 1) with a range of different infection prevalences and reproductive numbers. While there is a wide range of potential outcomes possible, most scenarios result in schools reclosing within 60 days with high numbers (69–163) of true infected individuals at the time of closure, despite closure being prompted by the detection of only 10 cases in a school of 1000. The one noteworthy exception to this was the case of schools that have low prevalence of the disease and a low in-school R_0_, which would be expected to remain open for approximately four months without seeding large disease clusters.

**Table 1:** Example scenarios for a range of hypothetical settings corresponding to currently relevant rates of prevalence and transmission.

Setting			Outcome	
Institution type	Prevalence	Testing?	Days to Closing	Number Infected at Close
K-12	high	no	17 (13–22)	163
K-12	average	no	27 (20–36)	135
K-12	low	no	46 (35–60)	127
K-12	high	yes	25 (15–44)	81
K-12	average	yes	54 (32–112)	72
K-12	low	yes	114 (69—NaN)	69
University	average	yes	37 (22–70)	691
University	average	no	20 (14–27)	1074

In the absence of widespread testing to reduce transmission and very low prevalence, most schools will have to shut down within 60 days. Despite closing being triggered by detection of only 10 cases in a school of 1000, the true number of infections seeded into the community at the time of closure ranges between 69–163 infections. The prevalence conditions correspond to high: 25 per 1000 (15 and 50 lower and upper bounds), average: 5 per 1000 (3 and 10 lower and upper bounds), low: 0.5 per 1000 (0.3 and 1 as lower and upper bounds). For schools without testing, we assumed an R_0_ = 3 (lower and upper bounds of 2.5 and 3.5), and for schools with rapid widespread testing we assumed an R_0_ = 1.5 (lower and upper bounds of 1.2 and 1.8).

As a sanity check, we also examined model predictions for the simulated scenario that would correspond to that of large state universities with students drawn from across the country. Our model predicts the rapid emergence of large clusters of COVID-19 in this setting.

**Fig 3:**
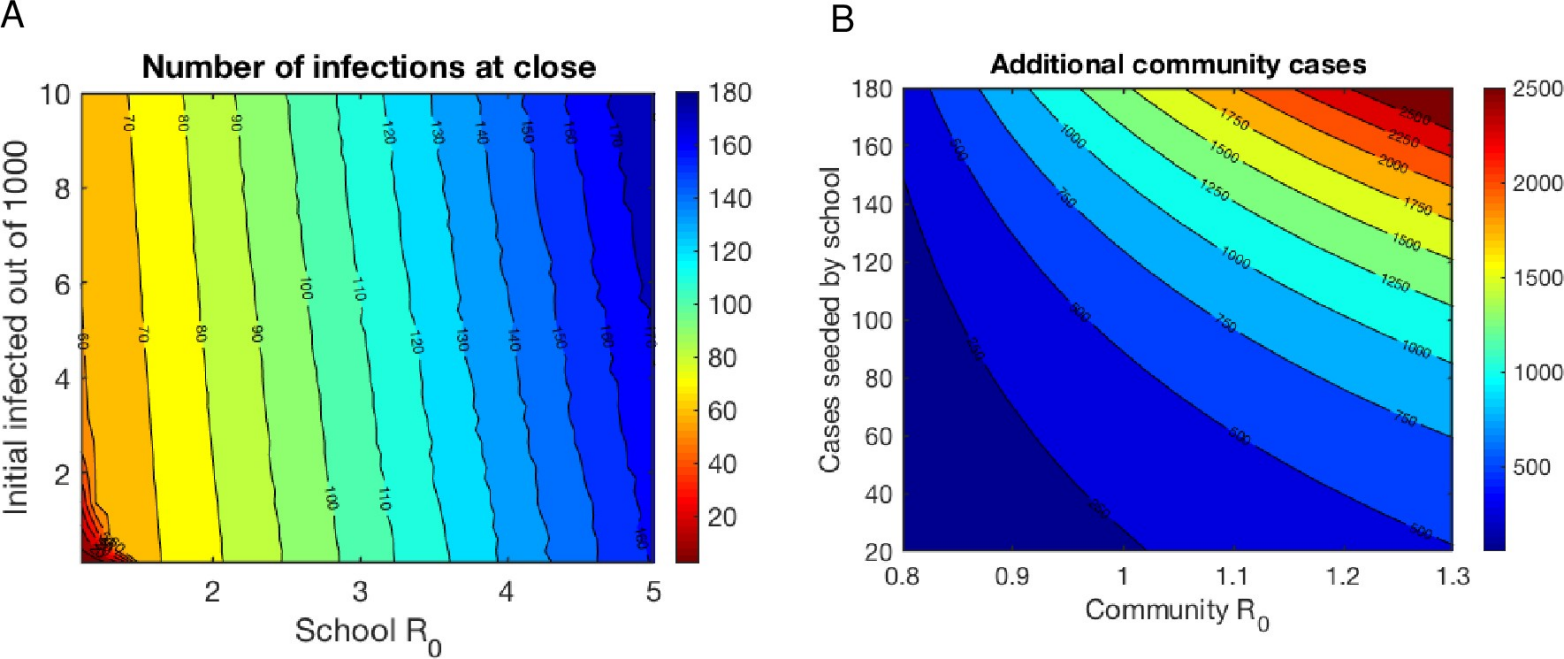
Even schools that close have the potential to seed infections in their community. A. Number infected at time of school closure as a function of initial prevalence and school R_0_, indicating even if a school closes when 1% of the student body has become a detected case, the true number of infections seeded into the community at that time could be anywhere from 2-18x greater than what was reported. B. Additional community cases in the next 100 days in a population of 50,000 as a function of infections seeded by school and a community’s R_0_, indicating the extreme risk for significant secondary infections into the community.

Once a cluster is initiated within a school population, it is unlikely that closing the school will end the transmission chains. Robust contact tracing for populations in close quarters is challenging, and the record so far indicates that quarantine and contact tracing protocols have been implemented in a haphazard manner [77–79]. Even students who are behaving safely after school closure are still interacting with their household members, presumably without PPE, potentially creating small family clusters of the disease. Thus it is reasonable to assume that a cluster of cases will continue to spread within the community. To examine the potential effect of school reopenings on community infections, we assumed that in-school infections would be seeded into communities at the time of school closures (Fig 3A), and we assumed community reproductive numbers consistent with the R_t_s for the US as of September 2020 [80], ranging from R_0_ = 0.8–1.3. Using this simplified approach, we calculated the expected number of additional cumulative infections in a community of 50,000 that would be generated by the infection chains initiated by a school over the next 100 days (Fig 3B). Our results indicate that even at relatively low community reproductive numbers, cases seeded by schools can lead to a high number of secondary cases in the community, with estimates ranging from less than 500 to close to 2,500 additional infections.

Our modeling thus makes a specific prediction- that school reopening would be followed by a wave of COVID-19 cases, and that school closures would slow down or reverse this growth. To test the generalizability of this prediction, we compared the dates of school reopening and closing across a set of 20 countries across the globe (Supplemental Material). Two countries kept their schools open for the entire duration of the pandemic, while one kept schools closed for the entire duration. In the remaining countries, the case count curves show dramatic rises following a lag period after school reopening, and after schools close again, case counts continue to rise for a while before beginning to fall. In 16 of the 17 countries that reopened their schools, there was at least one spike of COVID-19 cases within 4–8 weeks of school reopening. Of the 12 countries that closed their schools again after reopening, 11 showed a reduction in case counts within 4 weeks of school closing. While the first wave of COVID-19 cases happened in most countries even after schools were closed, the kinetics of school reopening and closing line up with COVID-19 case counts in a manner that is consistent with our model-based predictions.

## Discussion

The question of what will happen when schools reopen across the United States, in the face of sustained community transmission of SARS-CoV-2, is a pressing one. As the science around children’s susceptibility to SARS-CoV-2 infection and the mechanism of transmission of COVID-19 has evolved, a simple question to ask is: given what we know now, what is the impact of school reopening on disease spread?

The work described in this paper seeks to answer that question, using an epidemiological modeling framework that is updated with the current state of knowledge. We varied in-school R_0_ and local prevalence across plausible ranges and modeled both the immediate downstream and the longer-term consequences of school reopening.

There are a number of limitations to our modeling approach. First, we did not consider cohort structure among students. We made this choice because in a cohort setting, the choices that children make when they are not at school will drive a large fraction of the community and in-school transmissibility [81, 82]. Second, we assumed a minimal impact of symptomatic isolation. We made this choice based on previous findings that (given the kinetics of infectiousness of SARS-CoV-2), waiting even for a day after the appearance of symptoms largely degrades the utility of symptomatic isolation [64, 65]. Third, we did not explicitly model the effects of specific interventions (such as masks and surface cleaning), although our modeling addressed this implicitly in the R_0_ that was used (see Supplement for an in-depth discussion of the estimation of R_0_ in school settings). Fourth, our modeling of the dynamics of SARS-CoV-2 was fairly simplified- we did not account for the overdispersion in the transmission of SARS-CoV-2 and we assumed a constant rate of recovery and progression from latency to infectiousness for SARS-CoV-2. We made these choices in the interests of model parsimony, as our focus was on understanding the larger emergent behaviors without intervention at specific times, rather than predicting the kinetics of specific outbreaks at a granular level. Fifth, we assumed that infections in schools would occur rapidly compared to those in the community, spurring school closures and acting to seed infections into communities, rather than continually drive and amplify infections. This simplified scenario was chosen under the assumption that schools would rapidly close as cases were detected, with the intention to explore the consequences of these brief reopenings. The goal of our modeling was not divination (foreseeing the future) in all possible scenarios. Instead, we make a specific prediction regarding the downstream impact on schools and communities if schools reopen without adequate contact tracing and surveillance testing.

Our findings suggest that, for many communities within the United States, clusters will form quickly within schools upon reopening. This has been observed with colleges, although to some extent college outbreaks have been portrayed as a result of individual failings of college students, rather than as a structural consequence of prolonged indoor contact among large numbers of people in environments where the virus continues to spread (see S4 Table in S1 File for further examples of indoor spread).

Our findings also suggest that, given the higher proportion of asymptomatic cases among children, the detected cases will conceal a far larger burden of infection within the school setting. In examining the range of prevalence and reproductive numbers that allows for safe reopening, we find that there is a window where schools can reopen for up to 100 days without the emergence of a disease cluster exceeding a cumulative 1% of the student body with detected cases: with community prevalences below 1 in 1000 and in-school R_0_s below 1.5 (Fig 2C). As to whether schools will be able to meet these standards with the available safety measures is an open question. In reality, many schools may be unable to provide enough space, PPE, sanitization to meet the CDC’s guidelines. In the absence of surveillance testing, schools will rely on symptomatic isolation, which we and others have shown to be ineffective at controlling disease spread [64, 65]. More worryingly, there has been no evidence-based assessment of the efficacy of the current CDC guidelines in preventing disease spread.

Finally, our results suggest that school clusters, once initiated, have the potential for seeding enormous transmission chains within their communities. Given the high proportion of asymptomatic cases within children, and the current testing guidelines, these clusters may spread within the community for a considerable period of time before they are detected.

As a purely practical matter, our work has implications for successful school reopening. First, it points to the importance of taking measures to estimate the in-school prevalence of infections as early as possible. Rapid and widespread testing in general plays a key role in reducing transmission as a number of model-based analyses have shown very effectively [56, 83–85]. To this end, widespread deployment of mandatory or random surveillance testing (using antigen, saliva-based or LAMP testing) in schools may in fact be the key to safe school reopening. Rapid detection and isolation of new infections is critical in keeping schools open safely. Both in-school detected caseloads and county-level disease prevalence should be factored into decisions to reopen and re-close schools.

Second, it is critical that guidelines for limiting indoor spread of the virus be updated by public health authorities in an evidence-based manner [86], with a reassessment of the risks posed by possible aerosol and fecal-oral transmission. Beyond handwashing, mask wearing and physical distancing, there are a number of measures that schools can take to reduce R_0_ from its initial baseline- for example improved ventilation, widespread surveillance testing, contact tracing-based isolation and cohorting/podding to reduce time in the classroom (the hybrid model). Widespread surveillance testing and rigorous contact-tracing based isolation are not being broadly implemented in K-12 schools across the US, even though they have demonstrated their utility in other countries. However, many schools within the US have adopted some of these additional measures, in particular improving ventilation and reducing in-person time via the hybrid model. It is worth noting that the impact of the hybrid model is likely to be dependent on what students do on the days that they are not at school. In such a case, if students socialize on their days at home, or go to a daycare, the hybrid model may in fact not result in limiting transmission. Mapping the impact of specific measures to changes in the R_0_ Is beyond the scope of this work, but a number of other papers have explored this question, using both retrospective analyses of contact-tracing data [87] and agent-based simulations [88, 89]. We urge school authorities to implement as many measures as possible to reduce the in-school R_0_, as this variable has a profound impact on the feasibility of school reopening. Mandating mask use, upgrading ventilation systems, cohorting students, requiring students to continue to social distance/stay home when not at school and implementing widespread surveillance testing (particularly if rapid, easily performed tests are available) are all interventions worth considering if feasible.

Although the work in this paper was narrowly focused on the United States (in part because the US made the decision to reopen schools in the face of widespread community transmission), the predictions made by our modeling are broadly generalizable. Our modeling predicted specifically that school reopening would be followed by a wave of COVID-19 cases in surrounding communities. These model predictions were consistent with observed epidemiological dynamics in nearly every country examined, across various different regions, in different seasons, and in a number of cases, more than once within the same country. Our work points to the need for further statistical analyses of the link between school reopening and COVID-19 case kinetics across a larger pool of countries.

Sustained community transmission of SARS-CoV-2 has prolonged the pandemic, forcing a set of hard choices at the community and individual levels. As the long-term nature of the pandemic becomes apparent, the debate around whether or not children should return to school has been framed as a choice between the health risks of COVID-19 specifically for children and the educational and social benefits of a return to school for children. A number of arguments can be raised against this framing- given that children are taught in schools by (and in most cases, live at home with) adults, the cost-benefit of a return to school cannot be examined for them in isolation. Critically, if children’s return to school spikes a chain of transmission that percolates into their families and communities, then the debate is being framed as a false dual choice. It is possible for children’s return to school to create a situation where they compromise their health and the health of their families and communities, while also leading to school shutdowns and depriving them of the benefit of in-person learning.

Our work suggests that school reopenings should be done with careful consideration paid to COVID-19 prevalence and measures to limit the in-school R_0_ as much as possible. If not, school reopenings will spawn undetected disease clusters, leading to an inevitable return to remote learning, and a long shadow of disease that spreads through our communities in the months following school closure.

## Supporting information

S1 File (DOCX)

S2 File (PPTX)
