# Supplementary material for: In the long shadow of our best intentions: Model-based assessment of the consequences of school reopening during the COVID-19 pandemic
Source: PLoS One. 2021 Mar 25;16(3):e0248509. doi: 10.1371/journal.pone.0248509 (PMC7993767; doi:10.1371/journal.pone.0248509)
Supplement: S1 File — (DOCX) [file pone.0248509.s001.docx]

**In the long shadow of our best intentions: model-based assessment of the consequences of school reopening during the COVID-19 pandemic**

**Supplementary Information**

| **Parameter** | **Symbol** | **Value** | **Reference** |
| --- | --- | --- | --- |
| Basic reproductive number: number of individuals an infectious individual infects on average | R_0_ | 2.5 individuals [2.2, 3.5] | (CDC, 2020a) |
| Daily transmission rate | β | Basic reproductive number divided by duration of infection (β𝛄) | Calculated from R_0_ |
| Duration of time exposed but not detectable or infectious | 1/ α | 3 days | (Lauer *et al.*, 2020) |
| Duration of infection | 1/ 𝛄 | 14 days | (He *et al.*, 2020) |
| Time from initial infection to seek testing if symptomatic | 𝝉_seektest_ | 2.3 days (before symptoms) + 0.7 delay to get tested = 3 days | (He *et al.*, 2020) |
| Time to receive test result | 𝝉_testdelay_ | 4 days | (Baum *et al.*, 2020) |

**S1 Table. Model parameters and references for SEIR model**


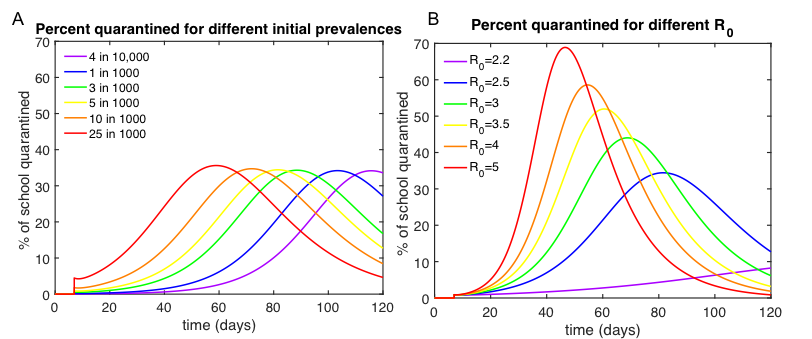


**S1 Fig. Estimated percent of school quarantined** at any time after school opening under A. varying number of people infected at the start at a constant R_0_=2.5 and B. varying school reproductive number at a constant initial prevalence of 5 in 1000.


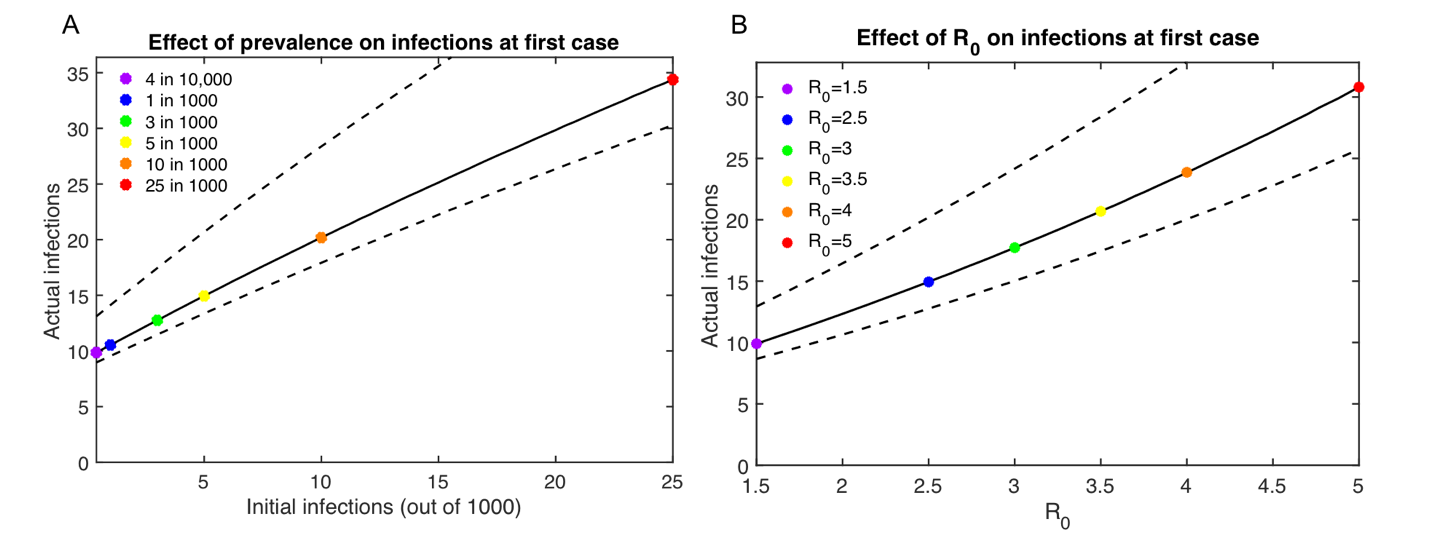


**S2 Fig.** Estimated number of actual infections at first detected case under A. varying initial number of infected people (in a school of 1000) at a constant R_0_=2.5 (lower and upper bounds represent R_0_= 2.2 and 3.5) and B. varying school reproductive number at a constant initial prevalence of 5 in 1000 (lower and upper bounds represent initial prevalence of 3 In 1000 and 10 in 1000).


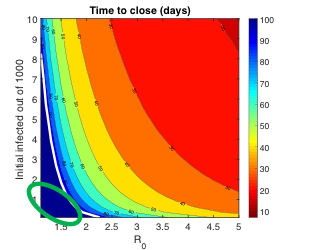


**S3 Fig.** Time to close as a function of school reproductive number and initial infection prevalence out of 1000, indicating that in order for schools to remain open for more than 100 days without 1% observed symptomatic infections, a school’s R0 must be below 1.5 and infection prevalence must be below 1 in 1000.

| **Location** | **NY Times cases per 100k in the last 7 days as of September 5^th^, 2020**  (The New York Times, 2020) | **Estimate of prevalence per 1000** |
| --- | --- | --- |
| Lafayette County, Mississippi | 609 | 30.5 (18.27-60.9) |
| Baldwin County, Georgia | 370 | 18.5 (11.1- 37.0) |
| Dallas County, Texas | 121 | 6.05 (3.63- 12.1) |
| Union County, New Jersey | 24 | 1.2 (0.72- 2.4) |
| Cheshire County, New Hampshire | 11 | 0.55 (0.33 – 1.1) |
| United States | 88 | 4.4 (2.6 – 8.8) |

**S2 Table. County prevalence values for different locations in the US**, assuming a 1 in 5 case reporting rate an infectious duration of 7 days. Upper and lower bounds of prevalence estimates are for a range of 1 in 10 and in 1 in 3 case reporting rate.

To estimate the prevalence per 1000 in a county, we take the cases per 100 thousand in the last 7 days from the New York Times website (The New York Times, 2020), following the methods outlined in recent work (Fox, Lachmann and Meyers, 2020). For example, in Dallas County, Texas there were 121 cases per 100 thousand in the last 7 days. We assume those represent all active infections. Then we assume that on average 1 in 5 infections are detected, and so the true infection prevalence is 5 times greater, yielding 605 infections per 100 thousand. We divide by 100 to get cases per 1000. Thus it is expected that in a school of 1000, about 6 individuals will show up infected in the first week of school (with a range of 4 to 12 depending on case reporting rate).

| **Scenario** | **Estimate of R_0_** | **Method of R_0_ estimation** | **Reference** |
| --- | --- | --- | --- |
| Wuhan, China prior to SARS-CoV-2 detection and lockdown | 1.4- 5.7 | Model-based inference | (Majumder and Mandl, 2020) (1.4 -4)  (Sanche *et al.*, 2020) (5.7 (3.8-8.9)) |
| Diamond Princess Cruise Ship | 14.8 | Model-based inference | (Rocklöv, Sjödin and Wilder-Smith, 2020) |
| Early epidemic in Europe and the US | 4.0-7.1 | Model-based inference | (Ke *et al.*, 2020) |

**S3 Table. R_0_ of COVID in different settings.** In settings where large indoor gatherings were still taking place, or where social distancing protocols were difficult to implement, baseline R_0_s for SARS-Cov-2 were considerably higher than the R_0_s in states within the US, which are currently around 1.

| **Scenario** | **Transmission reduction measures** | **Outbreak details** | **Reference** |
| --- | --- | --- | --- |
| Israel school reopening | masks required and windows opened but then a heat wave prompted relaxation and masks were removed and windows closed | Potential superspreading: 154 students and 26 staff members infected | (Kershner and Belluck) |
| SUNY Campus August 2020 | social distancing measures in place but student parties still occurred | More than 500 students positive in the first two weeks out of a school of 6,000 (where only 30 initial infections would have been expected) | (Ross, 2020) |
| Controlled cohort study on an 11 hour flight | symptomatic individuals screened and removed, N95 masks provided and worn except for meals and restrooms | One woman expected to have been infected from sitting 3 rows away from asymptomatic patient | (Bae *et al.*, 2020) |
| Outbreak in indoor choir in March 2020 in Seattle | no masks but at half- capacity | 2.5 hour choir practice- 52 of the 60 choir members became ill from single index patient | (Hamner *et al.*, 2020) |
| Outbreak in air-conditioned restaurant in Guangzhou, China in late January 2020. | Tables 1 meter apart | 9 others (4 from family of infected individual, 3 members from additional family and 2 members from another family) | (Lu *et al.*, 2020) |
| Outbreak in call center in South Korea in March 2020 | None- crowded with lots of talking | 94 employees on a the 11^th^ floor of the building, 216 were infected by a single index case | (Park *et al.*, 2020) |

**S4 Table. Documented indoor outbreaks with different levels of transmission reduction behaviors**

**References**

Bae, S. H. *et al.* (2020) ‘Asymptomatic Transmission of SARS-CoV-2 on Evacuation Flight’, *Emerging Infectious Disease journal*, 26(11). doi: 10.3201/eid2611.203353.

Baum, M. *et al.* (2020) ‘THE STATE OF THE NATION: A 50-STATE COVID-19 SURVEY Report #2’. doi: 10.31219/osf.io/j4kzb.

Fox, S. J., Lachmann, M. and Meyers, L. A. (2020) ‘Risks of COVID-19 Introductions as Schools Reopen Risks of COVID-19 Introductions as Schools Reopen’. Available at: https://sites.cns.utexas.edu/sites/default/files/cid/files/school_introduction_risks.pdf.

Hamner, L. *et al.* (2020) ‘High SARS-CoV-2 Attack Rate Following Exposure at a Choir Practice’, *Morbidity and Mortality Weekly Report High*, 69(19), pp. 606–610. Available at: https://www.cdc.gov/mmwr/volumes/69/wr/mm6919e6.htm.

Ke, R. *et al.* (2020) ‘Fast spread of COVID-19 in Europe and the US suggests the necessity of early, strong and comprehensive interventions’, *medRxiv : the preprint server for health sciences*. doi: 10.1101/2020.04.04.20050427.

Kershner, I. and Belluck, P. ‘When Covid Subsided, Israel Reopened Its Schools. It Didn’t Go Well’, *The New York Times*, August.

Lu, J. *et al.* (2020) ‘COVID-19 Outbreak Associated with Air Conditioning in Restaurant, Guangzhou, China, 2020’, *Emerging Infectious Disease journal*, 26(7), p. 1628. doi: 10.3201/eid2607.200764.

Majumder, M. and Mandl, K. D. (2020) ‘Early Transmissibility Assessment of a Novel Coronavirus in Wuhan, China’, *SSRN Electronic Journal*. doi: 10.2139/ssrn.3524675.

Park, S. Y. *et al.* (2020) ‘Coronavirus Disease Outbreak in Call Center, South Korea’, *Emerging Infectious Disease journal*, 26(8), p. 1666. doi: 10.3201/eid2608.201274.

Rocklöv, J., Sjödin, H. and Wilder-Smith, A. (2020) ‘COVID-19 outbreak on the Diamond Princess cruise ship: estimating the epidemic potential and effectiveness of public health countermeasures’, *Journal of Travel Medicine*, 27. doi: 10.1093/jtm/taaa030.

Ross, A. (2020) ‘A Few Students Threw Parties. Now an Entire SUNY Campus Is Shut Down’, *The New York Times*, 3 September.

Sanche, S. *et al.* (2020) ‘High Contagiousness and Rapid Spread of Severe Acute Respiratory Syndrome Coronavirus 2’, *Emerging Infectious Disease journal*, 26(7), p. 1470. doi: 10.3201/eid2607.200282.

The New York Times (2020) ‘Coronavirus in the U.S.: Latest Map and Case Count’, *The New York Times*, 5 September.
