# Supplementary material for: In the long shadow of our best intentions: Model-based assessment of the consequences of school reopening during the COVID-19 pandemic
Source: PLoS One. 2021 Mar 25;16(3):e0248509. doi: 10.1371/journal.pone.0248509 (PMC7993767; doi:10.1371/journal.pone.0248509)
Supplement: S2 File — (PPTX) [file pone.0248509.s002.pptx]

## Slide 1
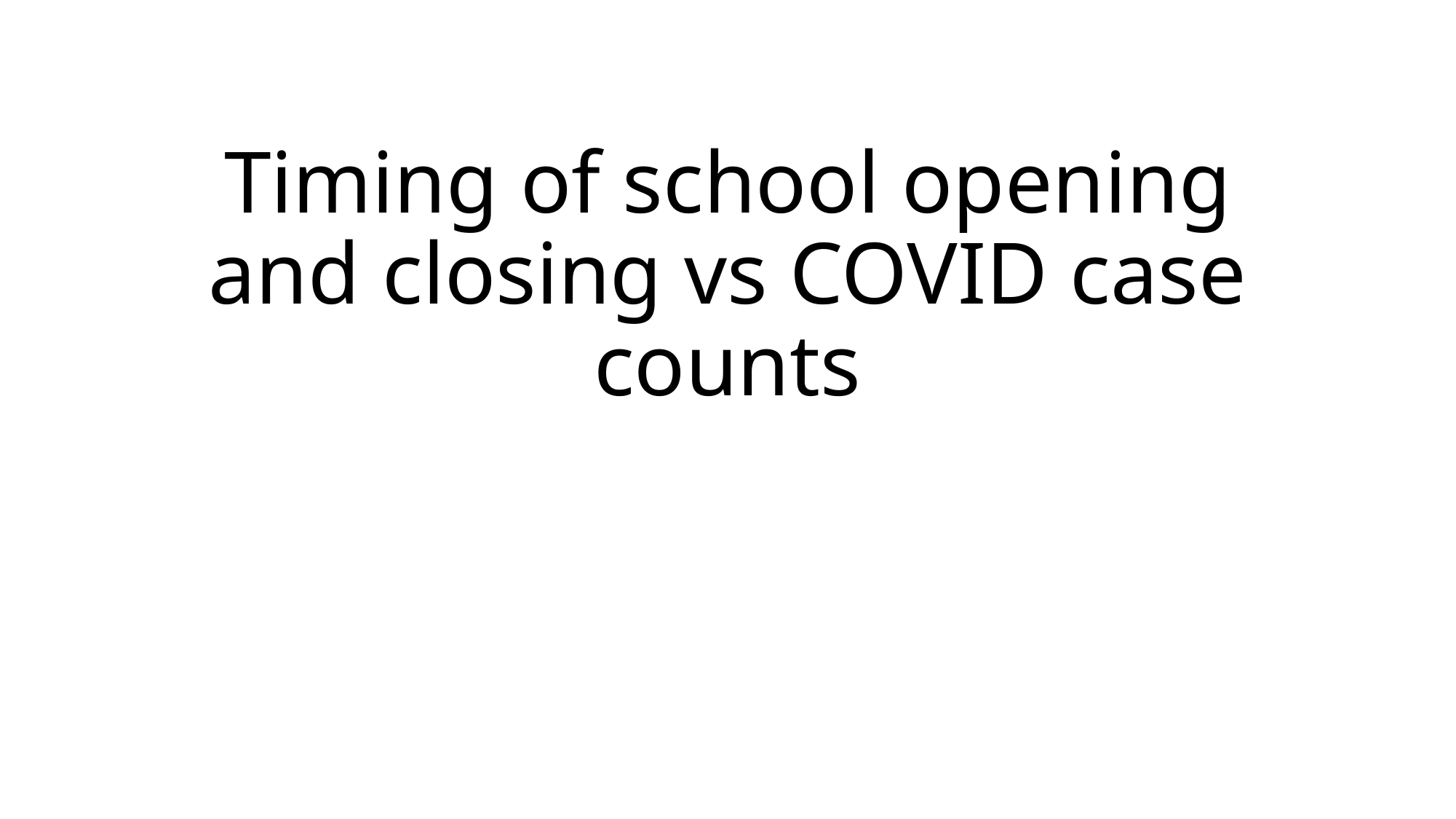

# Timing of school opening and closing vs COVID case counts

## Slide 2
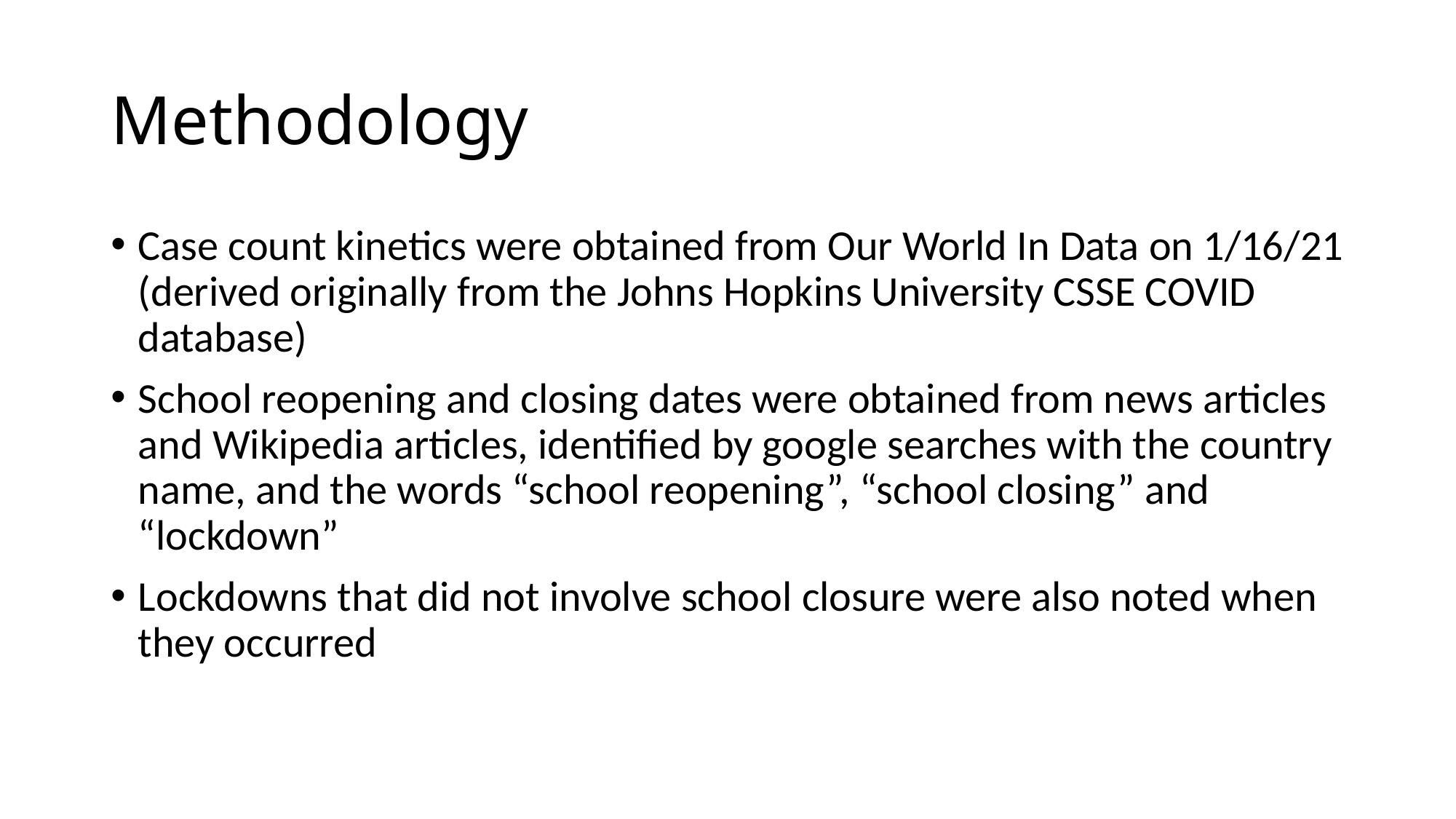

# Methodology
Case count kinetics were obtained from Our World In Data on 1/16/21 (derived originally from the Johns Hopkins University CSSE COVID database)
School reopening and closing dates were obtained from news articles and Wikipedia articles, identified by google searches with the country name, and the words “school reopening”, “school closing” and “lockdown”
Lockdowns that did not involve school closure were also noted when they occurred

## Slide 3
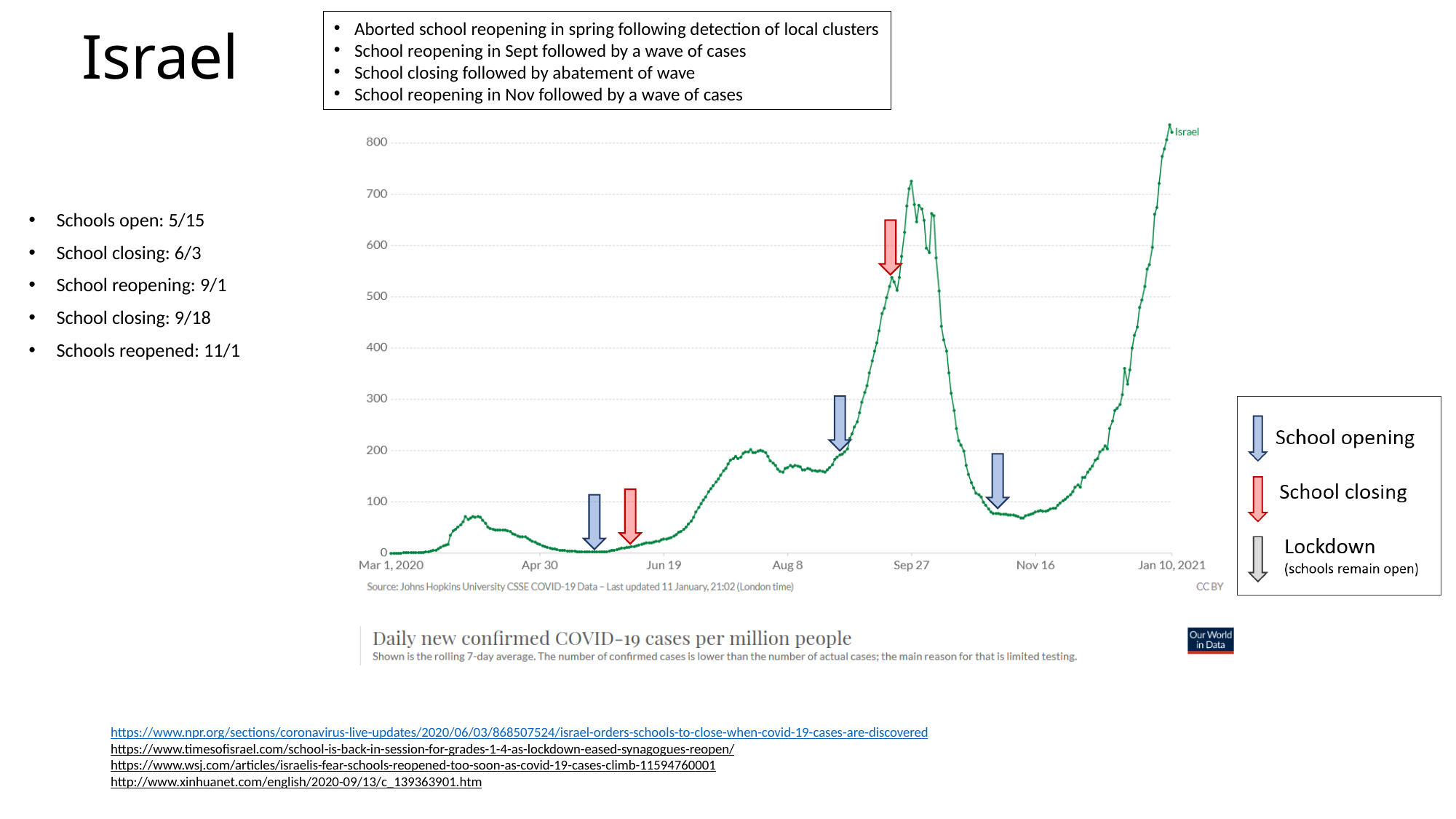

# Israel
Aborted school reopening in spring following detection of local clusters
School reopening in Sept followed by a wave of cases
School closing followed by abatement of wave
School reopening in Nov followed by a wave of cases
School opening
School closing
Lockdown
(schools remain open)
Schools open: 5/15
School closing: 6/3
School reopening: 9/1
School closing: 9/18
Schools reopened: 11/1
https://www.npr.org/sections/coronavirus-live-updates/2020/06/03/868507524/israel-orders-schools-to-close-when-covid-19-cases-are-discovered
https://www.timesofisrael.com/school-is-back-in-session-for-grades-1-4-as-lockdown-eased-synagogues-reopen/
https://www.wsj.com/articles/israelis-fear-schools-reopened-too-soon-as-covid-19-cases-climb-11594760001
http://www.xinhuanet.com/english/2020-09/13/c_139363901.htm

## Slide 4
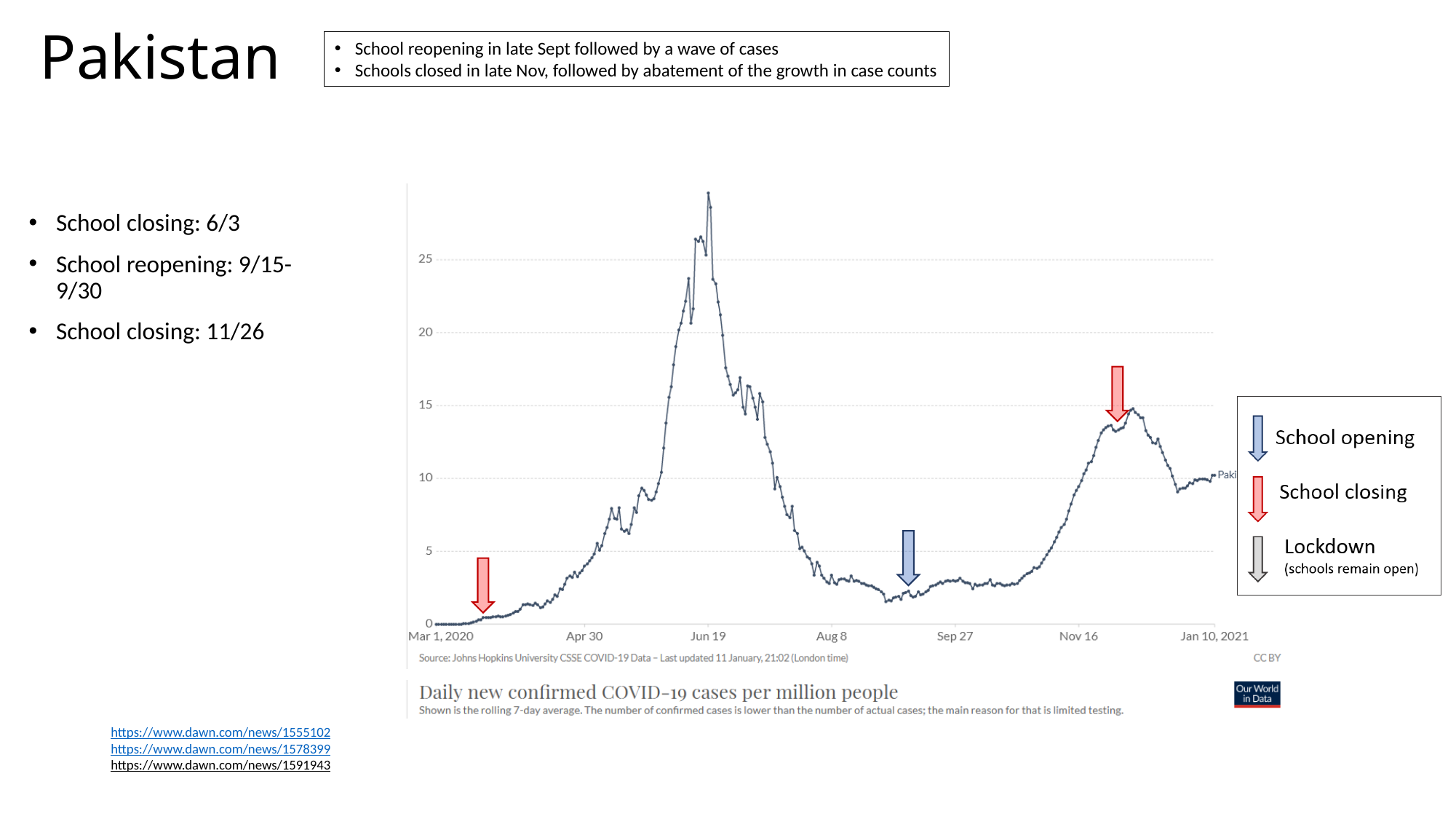

# Pakistan
School reopening in late Sept followed by a wave of cases
Schools closed in late Nov, followed by abatement of the growth in case counts
School closing: 6/3
School reopening: 9/15-9/30
School closing: 11/26
https://www.dawn.com/news/1555102
https://www.dawn.com/news/1578399
https://www.dawn.com/news/1591943

## Slide 5
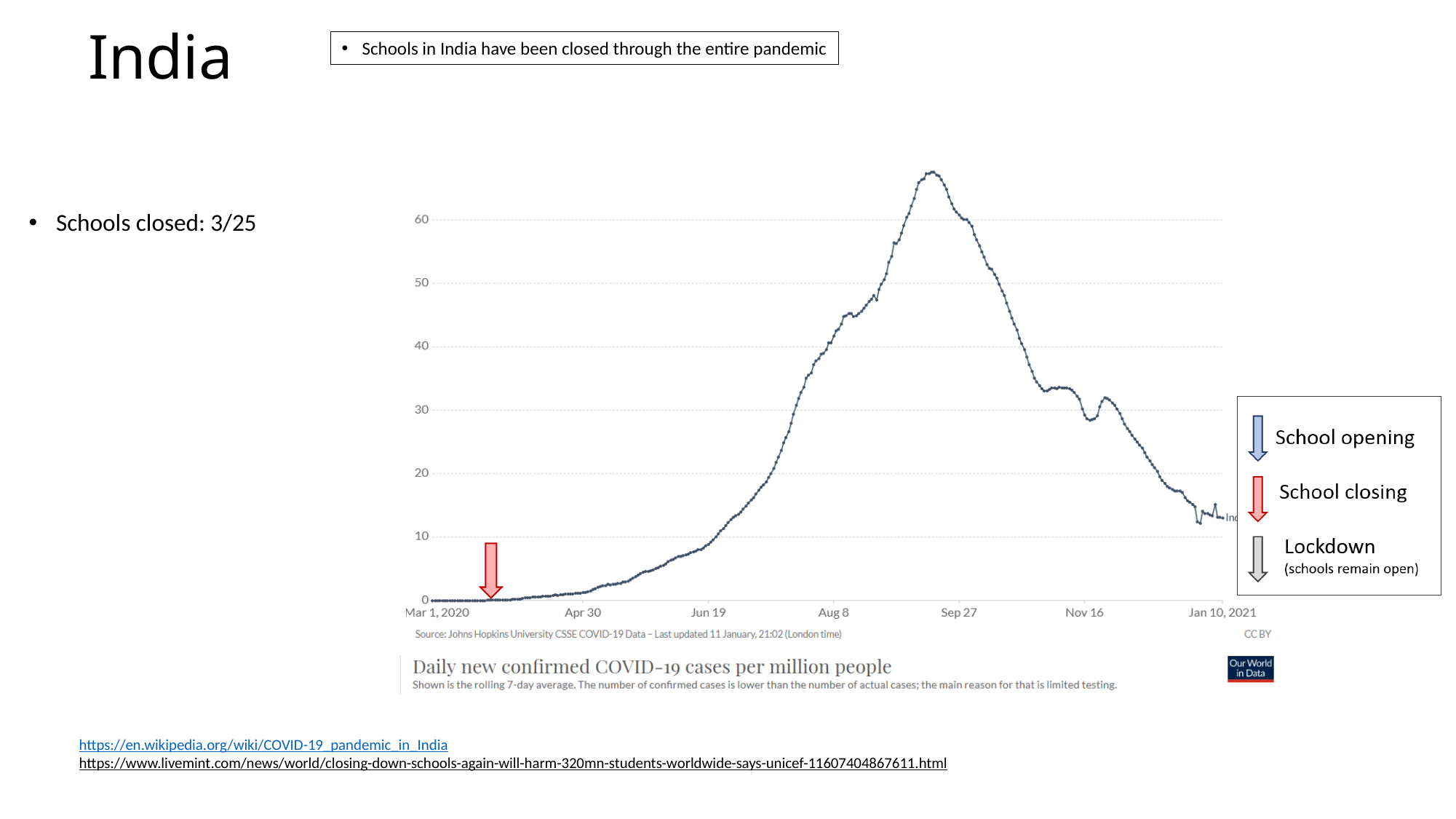

# India
Schools in India have been closed through the entire pandemic
Schools closed: 3/25
https://en.wikipedia.org/wiki/COVID-19_pandemic_in_India
https://www.livemint.com/news/world/closing-down-schools-again-will-harm-320mn-students-worldwide-says-unicef-11607404867611.html

## Slide 6
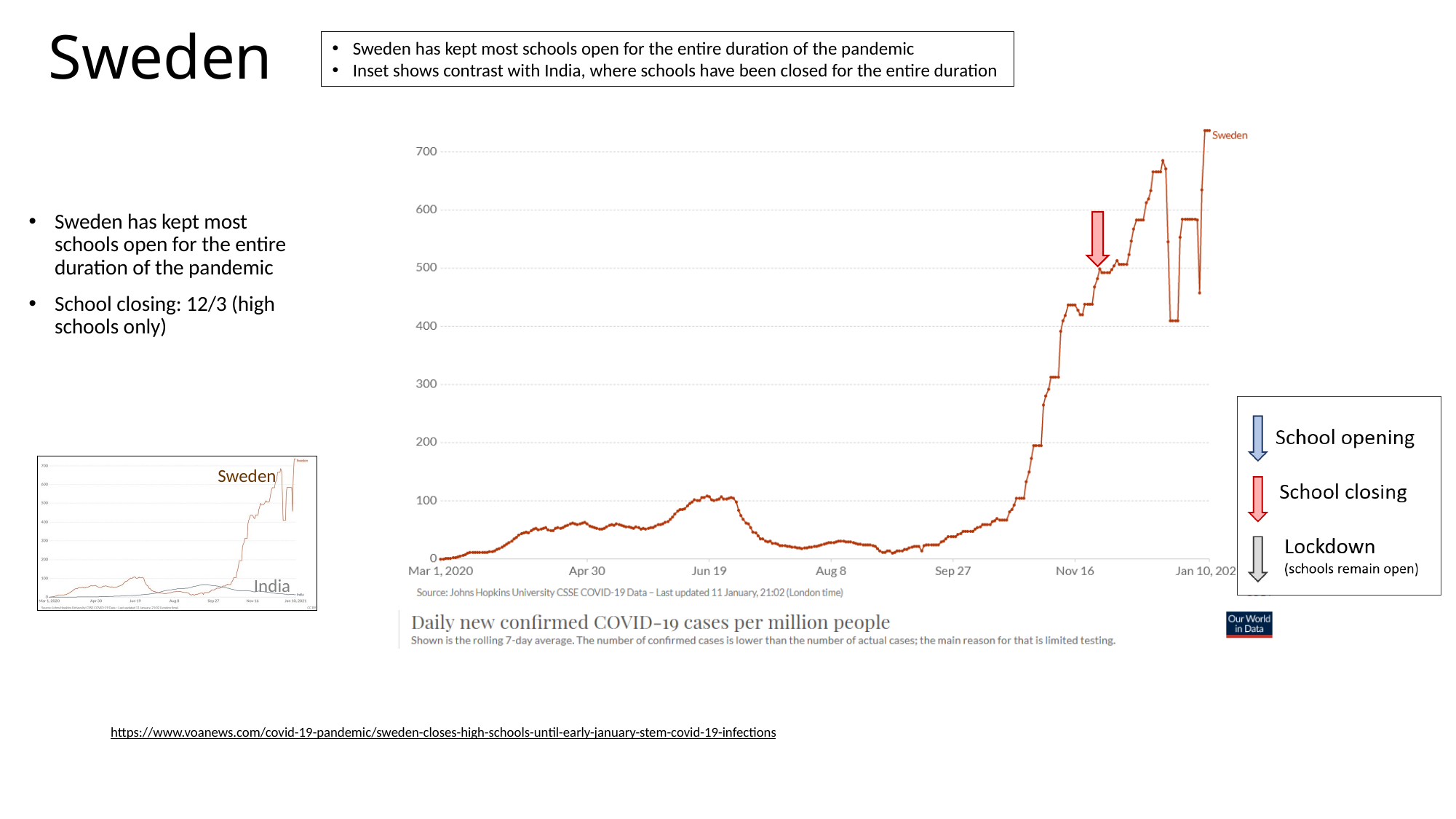

# Sweden
Sweden has kept most schools open for the entire duration of the pandemic
Inset shows contrast with India, where schools have been closed for the entire duration
Sweden has kept most schools open for the entire duration of the pandemic
School closing: 12/3 (high schools only)
Sweden
India
https://www.voanews.com/covid-19-pandemic/sweden-closes-high-schools-until-early-january-stem-covid-19-infections

## Slide 7
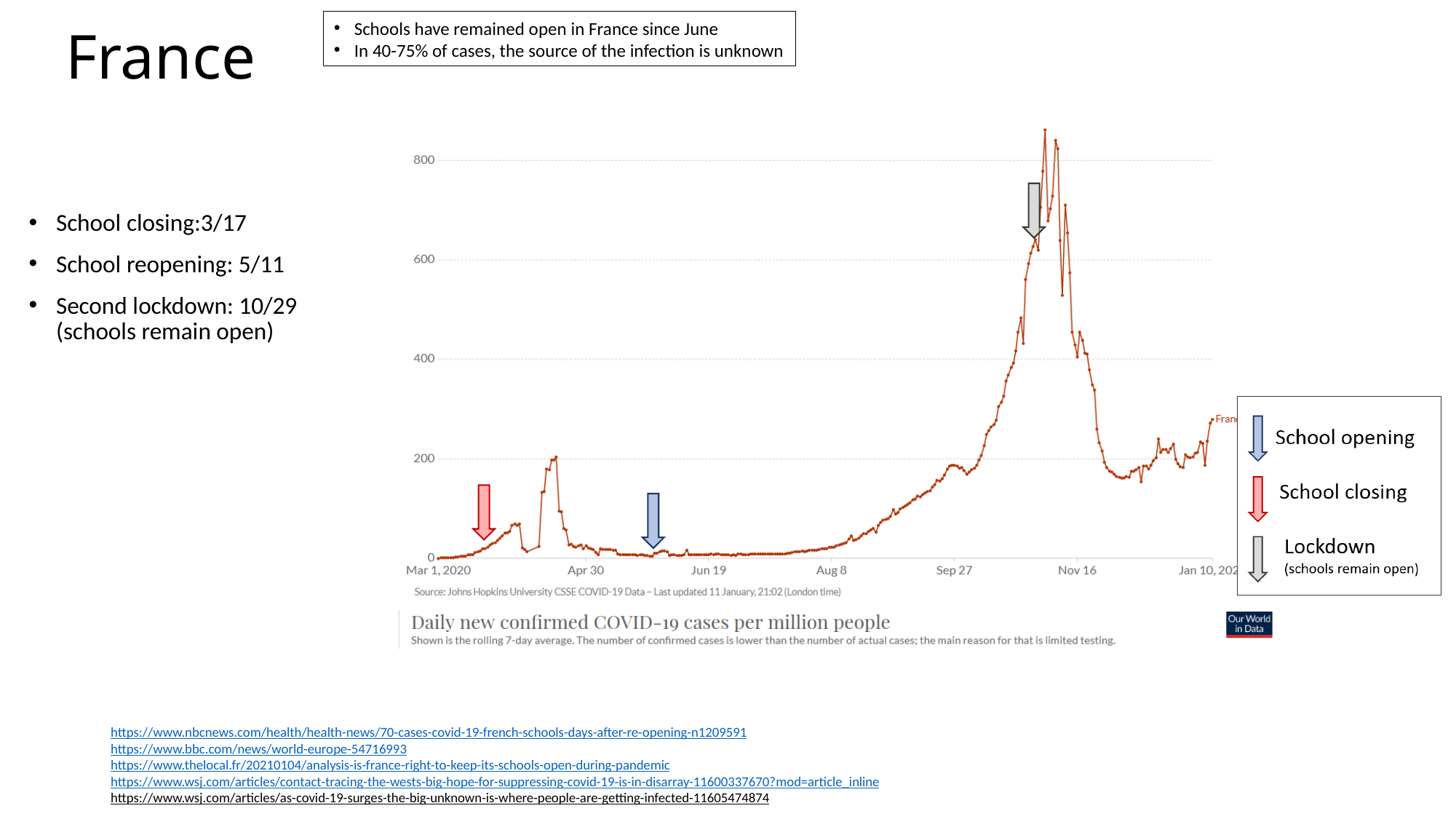

# France
Schools have remained open in France since June
In 40-75% of cases, the source of the infection is unknown
School closing:3/17
School reopening: 5/11
Second lockdown: 10/29 (schools remain open)
https://www.nbcnews.com/health/health-news/70-cases-covid-19-french-schools-days-after-re-opening-n1209591
https://www.bbc.com/news/world-europe-54716993
https://www.thelocal.fr/20210104/analysis-is-france-right-to-keep-its-schools-open-during-pandemic
https://www.wsj.com/articles/contact-tracing-the-wests-big-hope-for-suppressing-covid-19-is-in-disarray-11600337670?mod=article_inline
https://www.wsj.com/articles/as-covid-19-surges-the-big-unknown-is-where-people-are-getting-infected-11605474874

## Slide 8
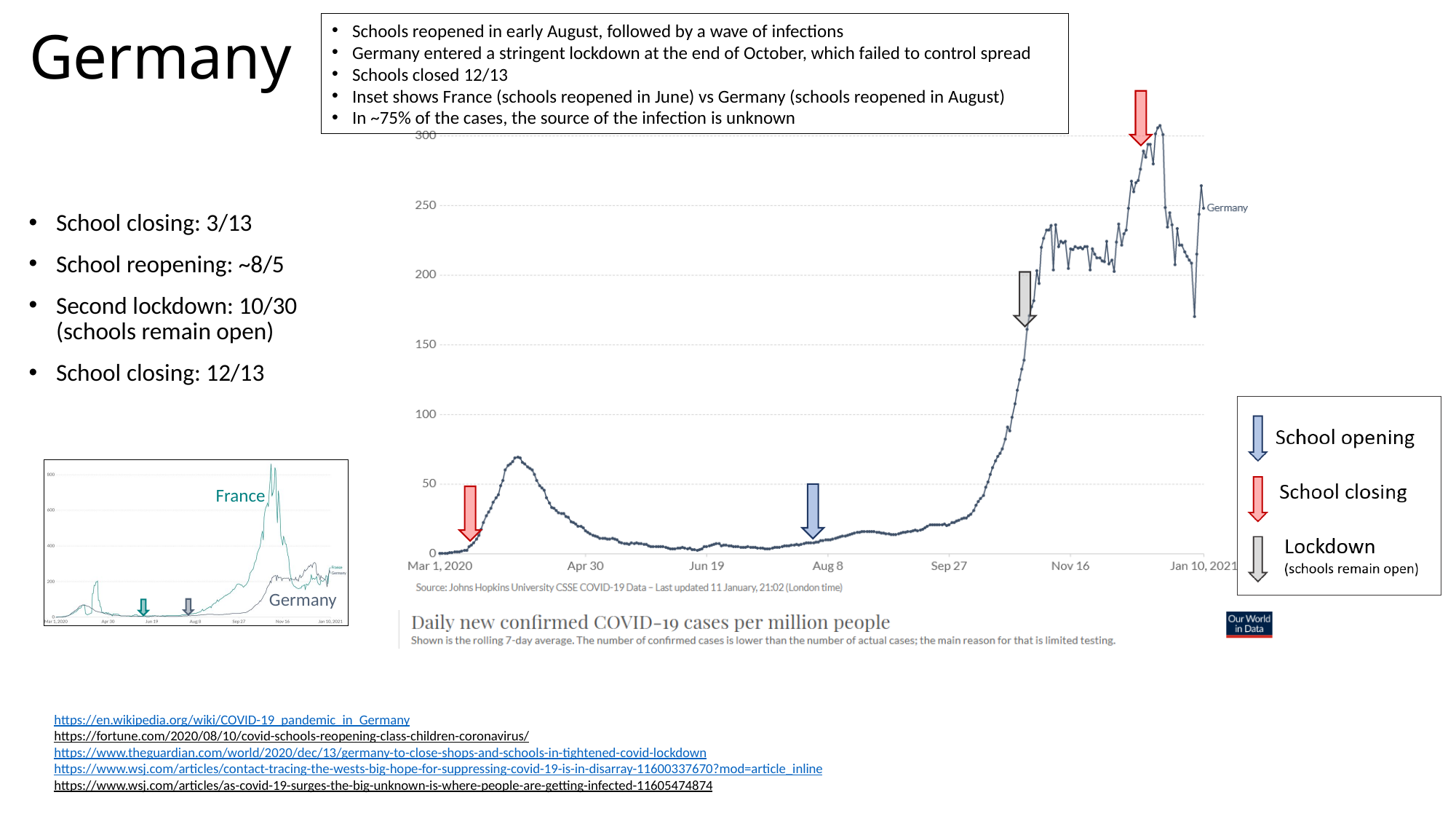

# Germany
Schools reopened in early August, followed by a wave of infections
Germany entered a stringent lockdown at the end of October, which failed to control spread
Schools closed 12/13
Inset shows France (schools reopened in June) vs Germany (schools reopened in August)
In ~75% of the cases, the source of the infection is unknown
School closing: 3/13
School reopening: ~8/5
Second lockdown: 10/30 (schools remain open)
School closing: 12/13
France
Germany
https://en.wikipedia.org/wiki/COVID-19_pandemic_in_Germany
https://fortune.com/2020/08/10/covid-schools-reopening-class-children-coronavirus/
https://www.theguardian.com/world/2020/dec/13/germany-to-close-shops-and-schools-in-tightened-covid-lockdown
https://www.wsj.com/articles/contact-tracing-the-wests-big-hope-for-suppressing-covid-19-is-in-disarray-11600337670?mod=article_inline
https://www.wsj.com/articles/as-covid-19-surges-the-big-unknown-is-where-people-are-getting-infected-11605474874

## Slide 9
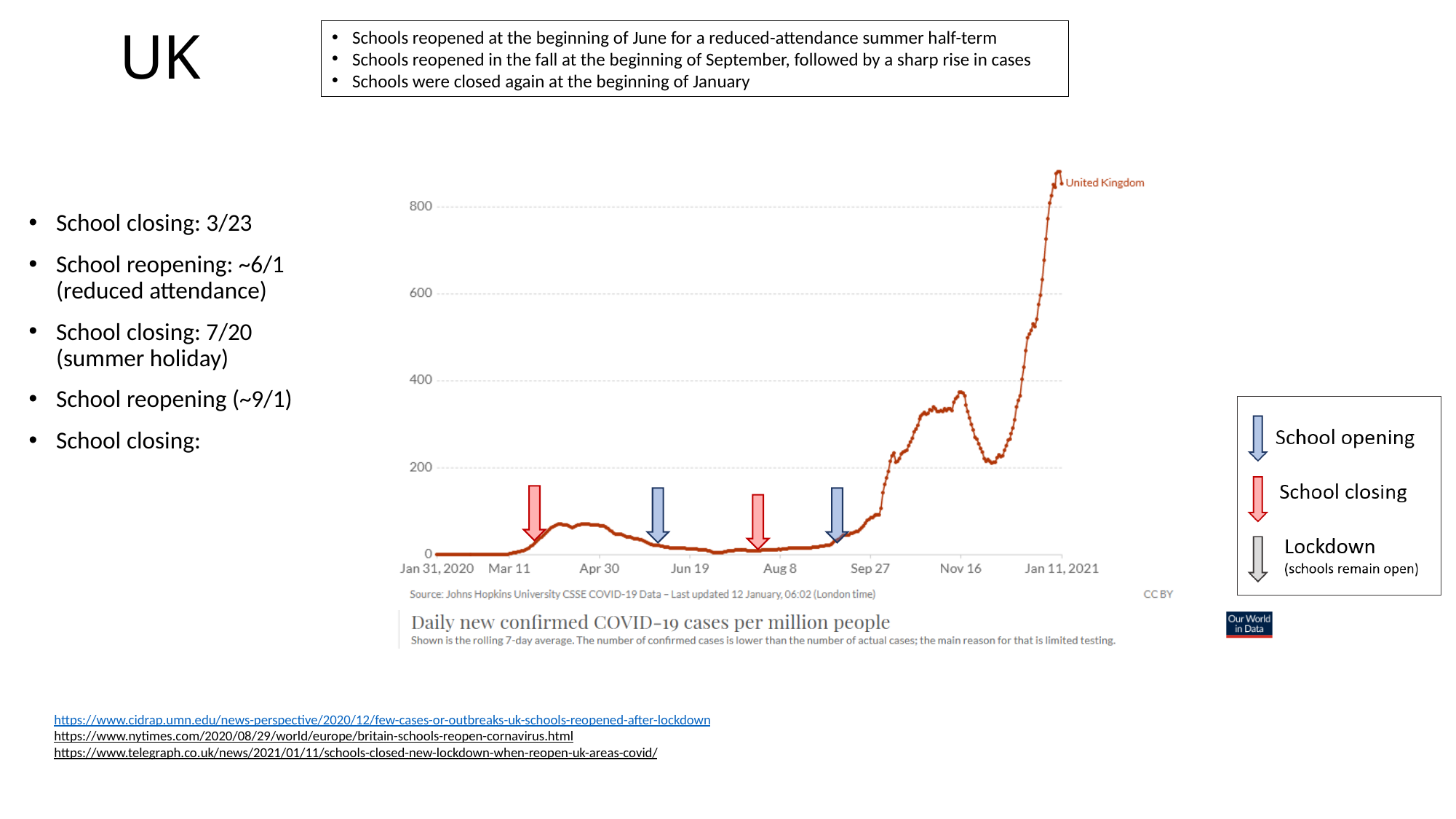

# UK
Schools reopened at the beginning of June for a reduced-attendance summer half-term
Schools reopened in the fall at the beginning of September, followed by a sharp rise in cases
Schools were closed again at the beginning of January
School closing: 3/23
School reopening: ~6/1 (reduced attendance)
School closing: 7/20 (summer holiday)
School reopening (~9/1)
School closing:
https://www.cidrap.umn.edu/news-perspective/2020/12/few-cases-or-outbreaks-uk-schools-reopened-after-lockdown
https://www.nytimes.com/2020/08/29/world/europe/britain-schools-reopen-cornavirus.html
https://www.telegraph.co.uk/news/2021/01/11/schools-closed-new-lockdown-when-reopen-uk-areas-covid/

## Slide 10
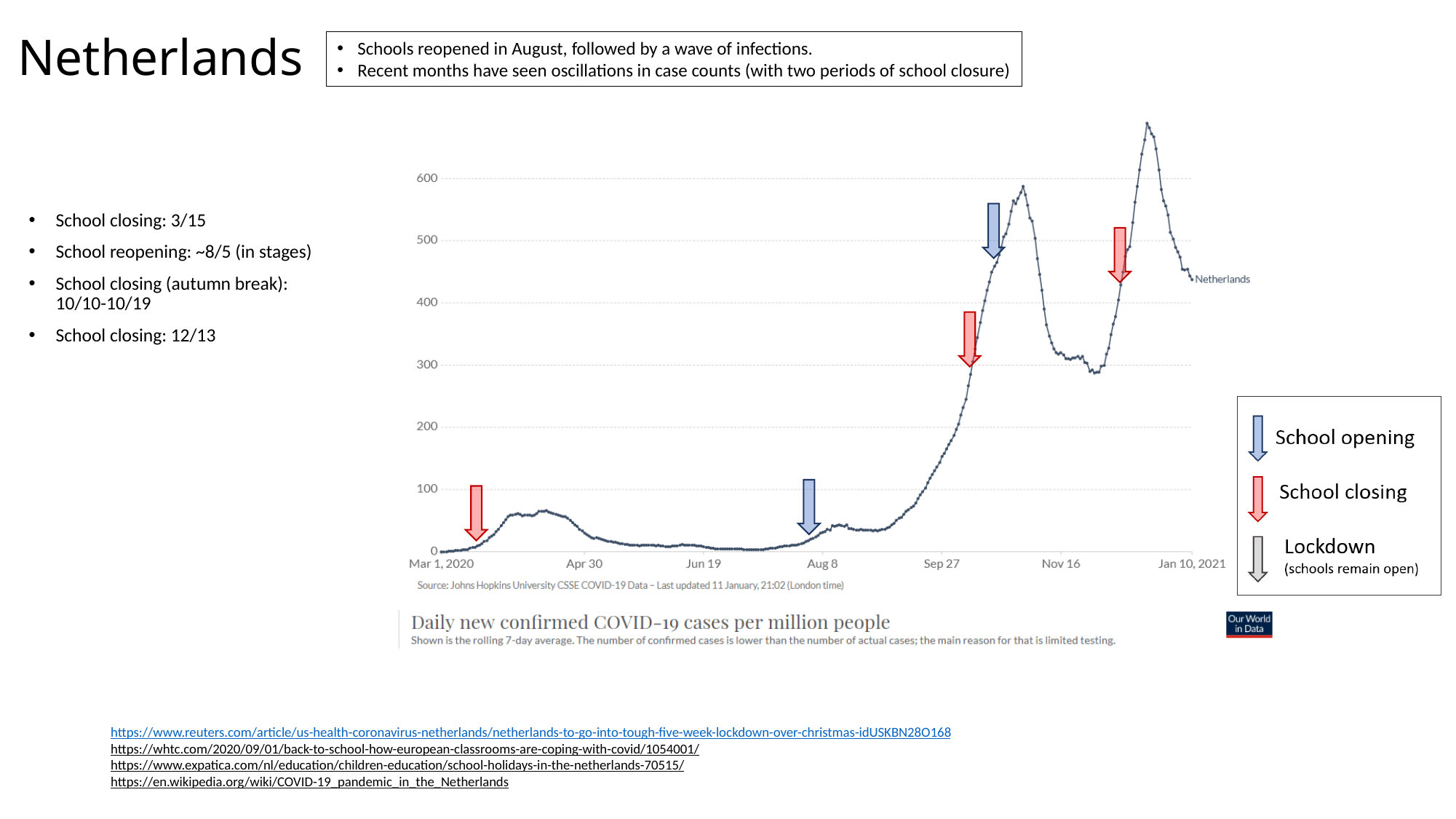

# Netherlands
Schools reopened in August, followed by a wave of infections.
Recent months have seen oscillations in case counts (with two periods of school closure)
School closing: 3/15
School reopening: ~8/5 (in stages)
School closing (autumn break): 10/10-10/19
School closing: 12/13
https://www.reuters.com/article/us-health-coronavirus-netherlands/netherlands-to-go-into-tough-five-week-lockdown-over-christmas-idUSKBN28O168
https://whtc.com/2020/09/01/back-to-school-how-european-classrooms-are-coping-with-covid/1054001/
https://www.expatica.com/nl/education/children-education/school-holidays-in-the-netherlands-70515/
https://en.wikipedia.org/wiki/COVID-19_pandemic_in_the_Netherlands

## Slide 11
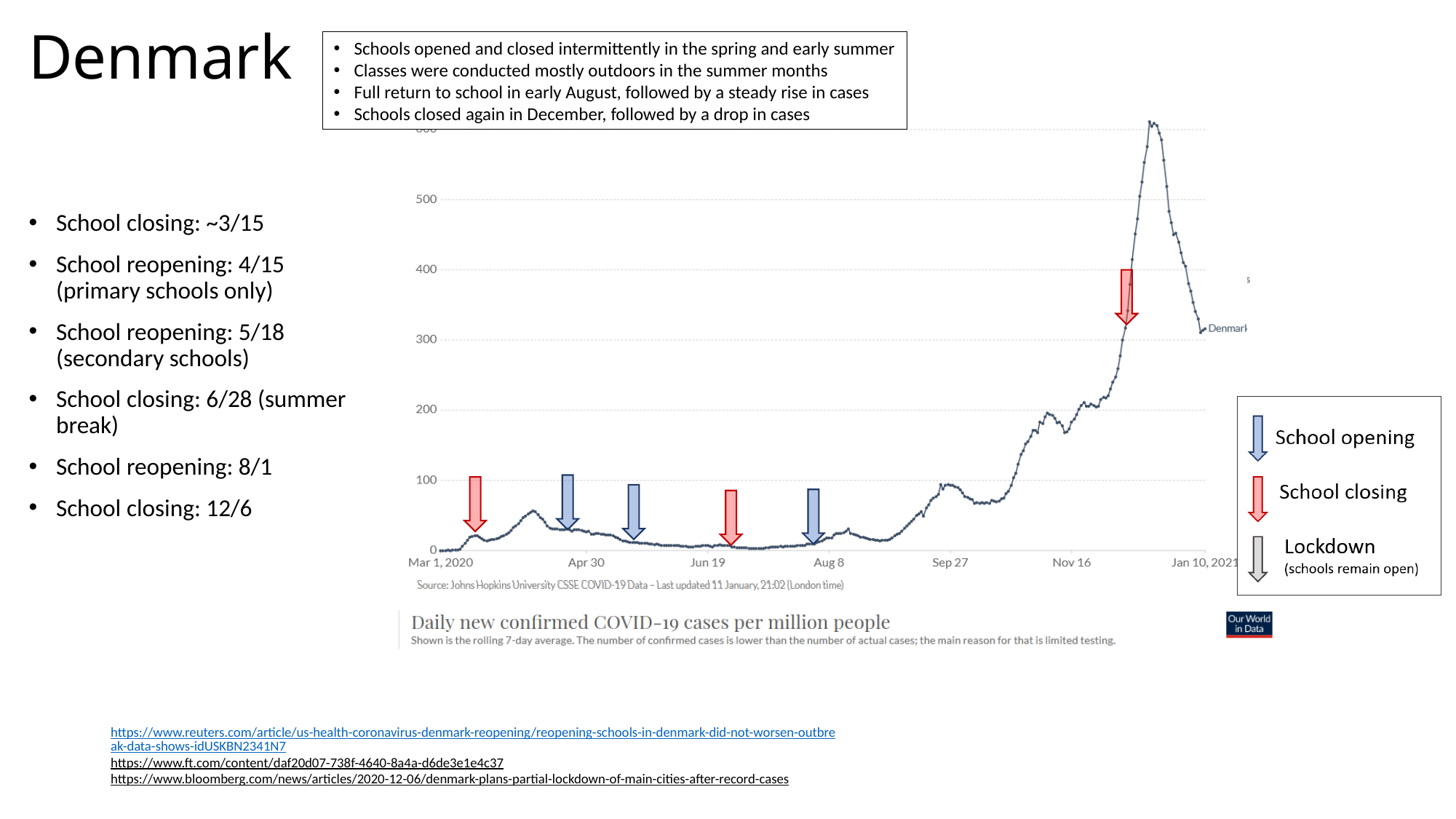

# Denmark
Schools opened and closed intermittently in the spring and early summer
Classes were conducted mostly outdoors in the summer months
Full return to school in early August, followed by a steady rise in cases
Schools closed again in December, followed by a drop in cases
School closing: ~3/15
School reopening: 4/15 (primary schools only)
School reopening: 5/18 (secondary schools)
School closing: 6/28 (summer break)
School reopening: 8/1
School closing: 12/6
https://www.reuters.com/article/us-health-coronavirus-denmark-reopening/reopening-schools-in-denmark-did-not-worsen-outbreak-data-shows-idUSKBN2341N7
https://www.ft.com/content/daf20d07-738f-4640-8a4a-d6de3e1e4c37
https://www.bloomberg.com/news/articles/2020-12-06/denmark-plans-partial-lockdown-of-main-cities-after-record-cases

## Slide 12
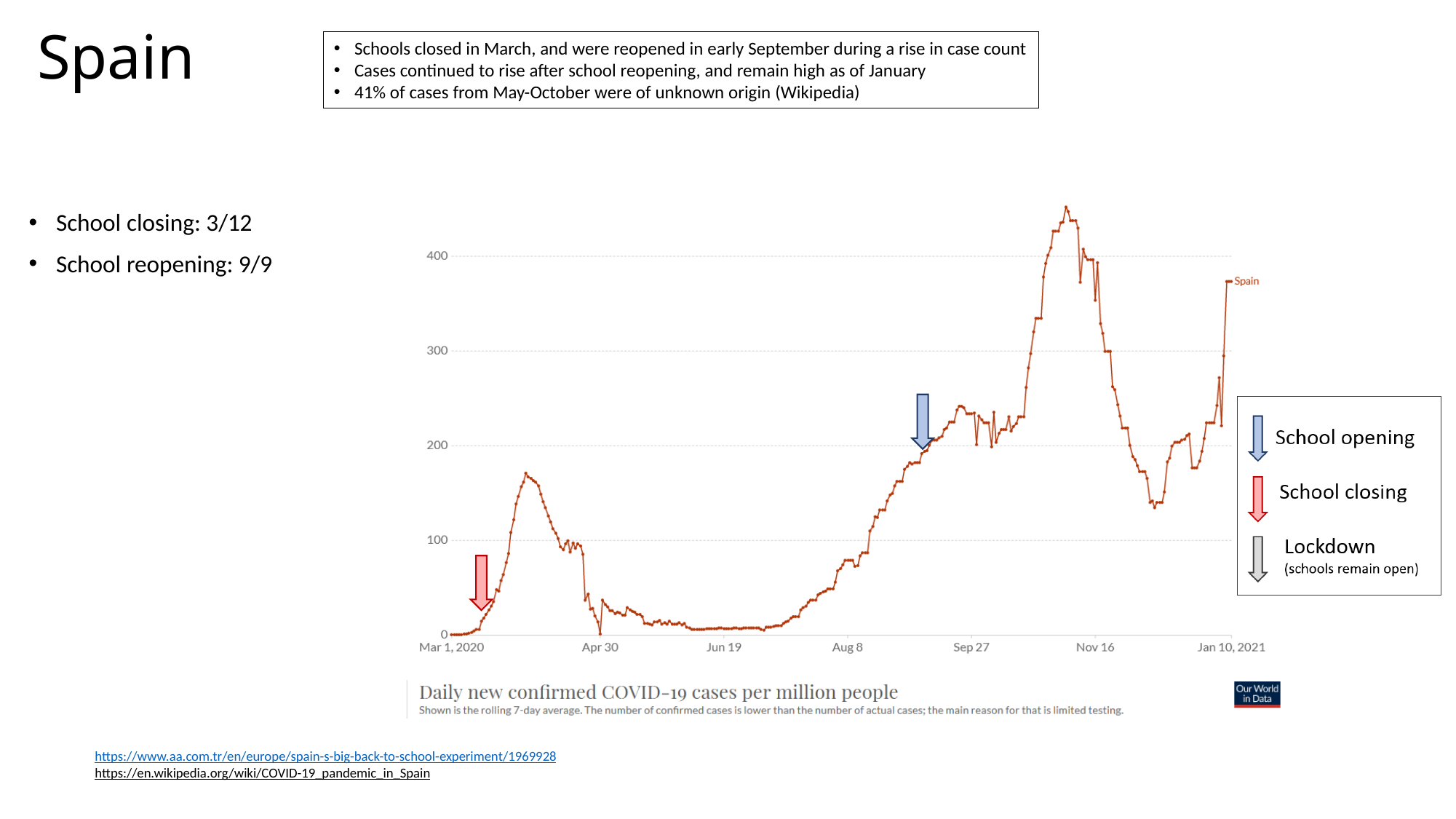

# Spain
Schools closed in March, and were reopened in early September during a rise in case count
Cases continued to rise after school reopening, and remain high as of January
41% of cases from May-October were of unknown origin (Wikipedia)
School closing: 3/12
School reopening: 9/9
https://www.aa.com.tr/en/europe/spain-s-big-back-to-school-experiment/1969928
https://en.wikipedia.org/wiki/COVID-19_pandemic_in_Spain

## Slide 13
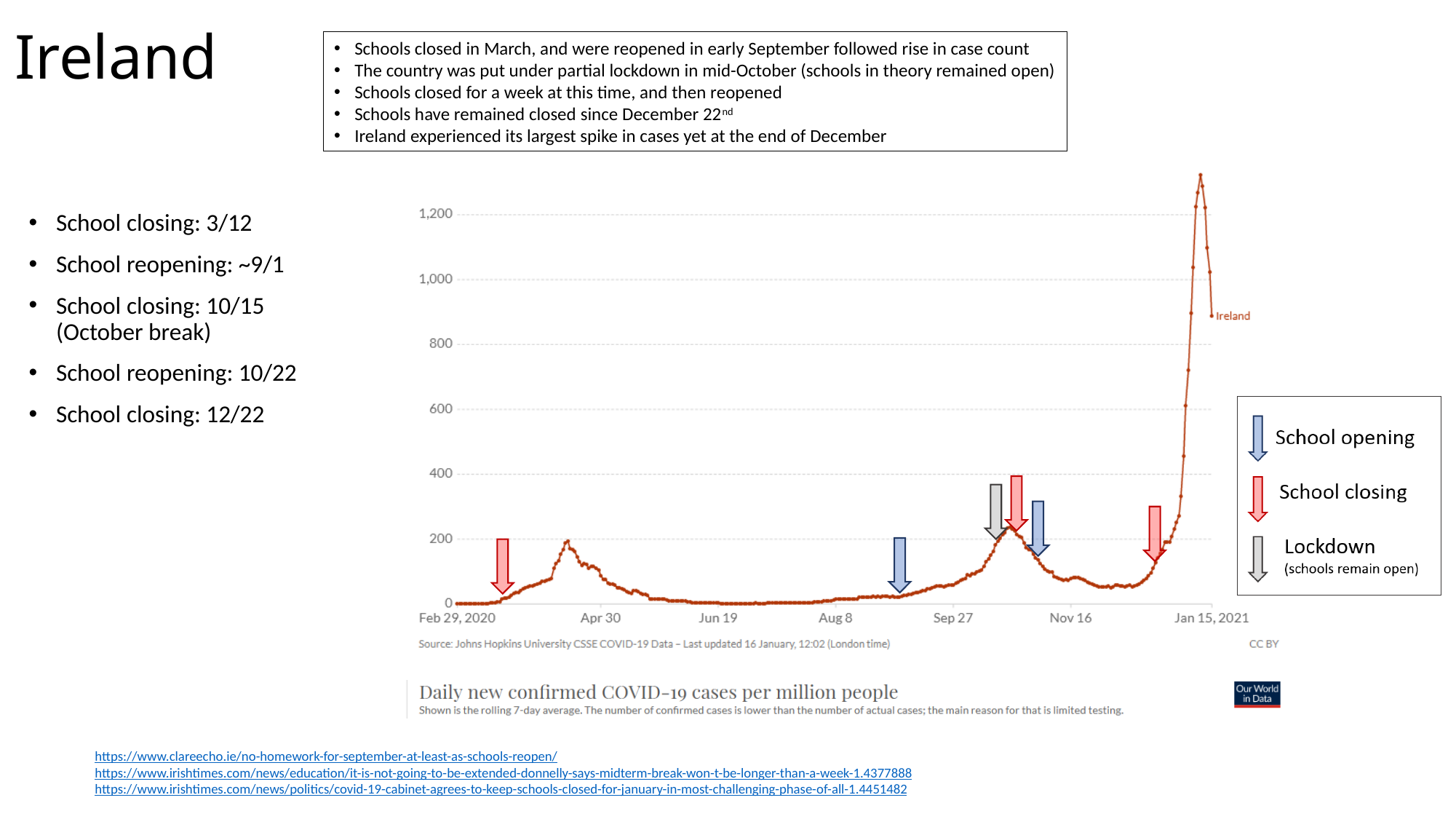

# Ireland
Schools closed in March, and were reopened in early September followed rise in case count
The country was put under partial lockdown in mid-October (schools in theory remained open)
Schools closed for a week at this time, and then reopened
Schools have remained closed since December 22nd
Ireland experienced its largest spike in cases yet at the end of December
School closing: 3/12
School reopening: ~9/1
School closing: 10/15 (October break)
School reopening: 10/22
School closing: 12/22
https://www.clareecho.ie/no-homework-for-september-at-least-as-schools-reopen/
https://www.irishtimes.com/news/education/it-is-not-going-to-be-extended-donnelly-says-midterm-break-won-t-be-longer-than-a-week-1.4377888
https://www.irishtimes.com/news/politics/covid-19-cabinet-agrees-to-keep-schools-closed-for-january-in-most-challenging-phase-of-all-1.4451482

## Slide 14
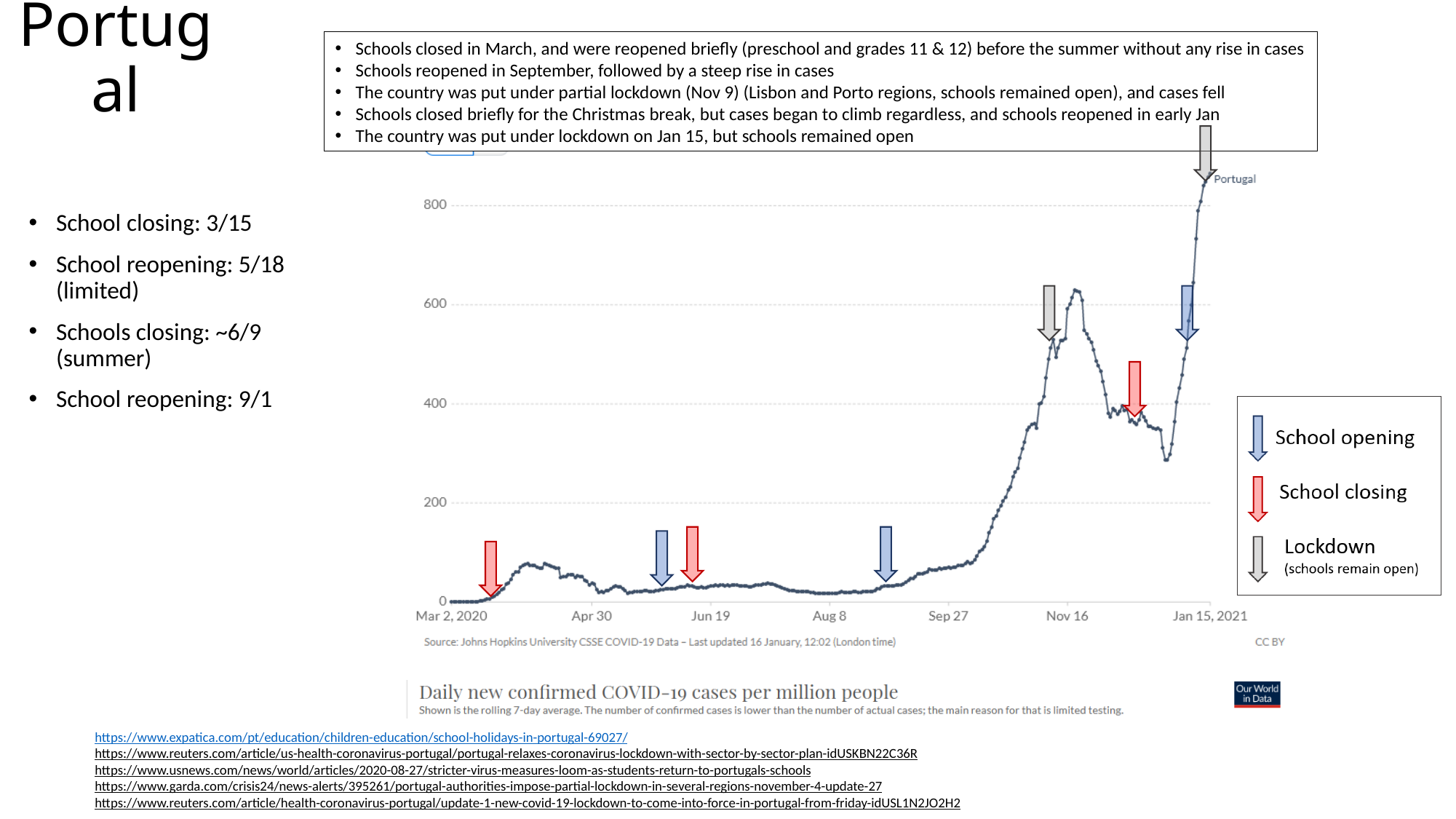

# Portugal
Schools closed in March, and were reopened briefly (preschool and grades 11 & 12) before the summer without any rise in cases
Schools reopened in September, followed by a steep rise in cases
The country was put under partial lockdown (Nov 9) (Lisbon and Porto regions, schools remained open), and cases fell
Schools closed briefly for the Christmas break, but cases began to climb regardless, and schools reopened in early Jan
The country was put under lockdown on Jan 15, but schools remained open
School closing: 3/15
School reopening: 5/18 (limited)
Schools closing: ~6/9 (summer)
School reopening: 9/1
https://www.expatica.com/pt/education/children-education/school-holidays-in-portugal-69027/
https://www.reuters.com/article/us-health-coronavirus-portugal/portugal-relaxes-coronavirus-lockdown-with-sector-by-sector-plan-idUSKBN22C36R
https://www.usnews.com/news/world/articles/2020-08-27/stricter-virus-measures-loom-as-students-return-to-portugals-schools
https://www.garda.com/crisis24/news-alerts/395261/portugal-authorities-impose-partial-lockdown-in-several-regions-november-4-update-27
https://www.reuters.com/article/health-coronavirus-portugal/update-1-new-covid-19-lockdown-to-come-into-force-in-portugal-from-friday-idUSL1N2JO2H2

## Slide 15
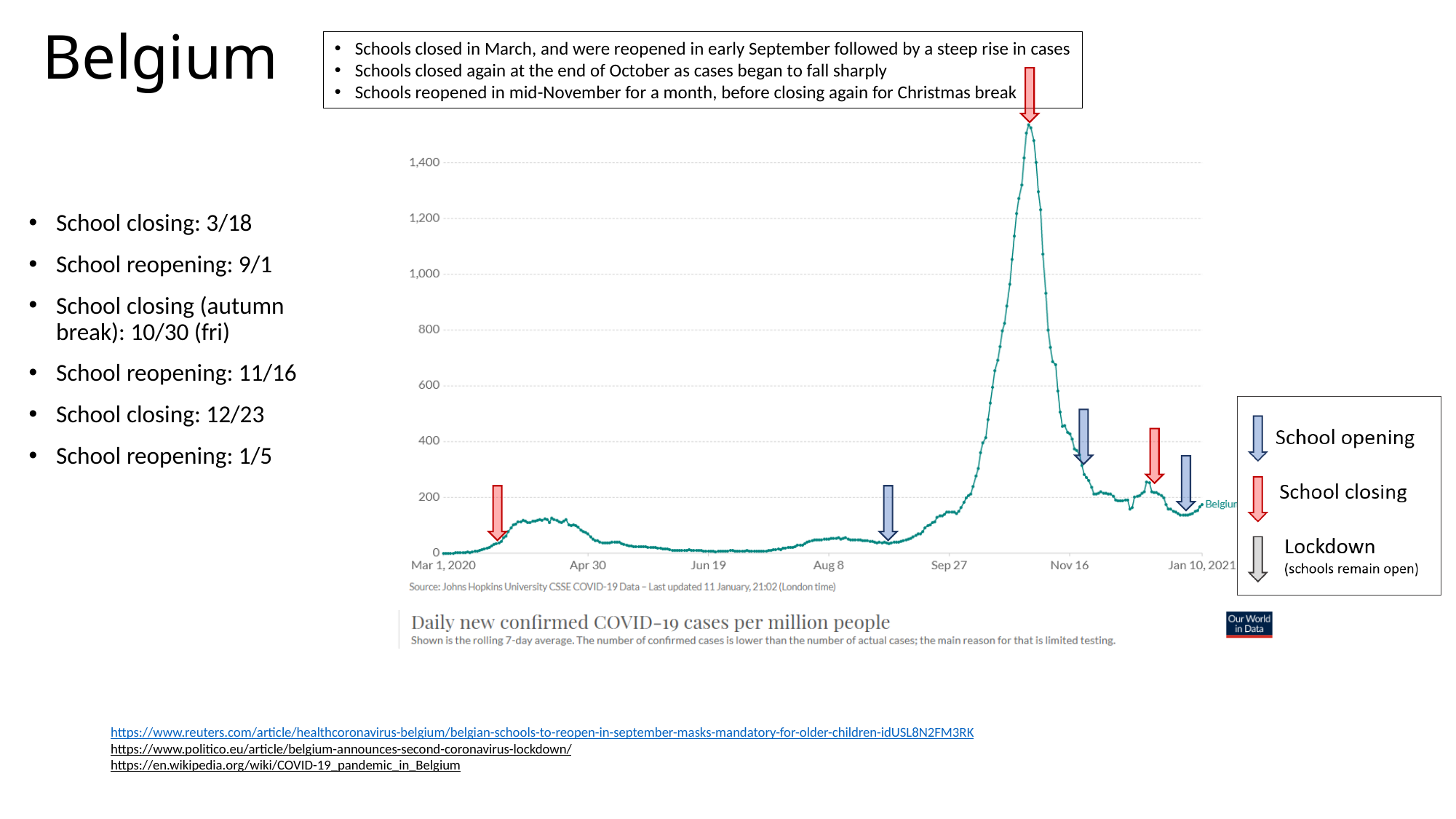

# Belgium
Schools closed in March, and were reopened in early September followed by a steep rise in cases
Schools closed again at the end of October as cases began to fall sharply
Schools reopened in mid-November for a month, before closing again for Christmas break
School closing: 3/18
School reopening: 9/1
School closing (autumn break): 10/30 (fri)
School reopening: 11/16
School closing: 12/23
School reopening: 1/5
https://www.reuters.com/article/healthcoronavirus-belgium/belgian-schools-to-reopen-in-september-masks-mandatory-for-older-children-idUSL8N2FM3RK
https://www.politico.eu/article/belgium-announces-second-coronavirus-lockdown/
https://en.wikipedia.org/wiki/COVID-19_pandemic_in_Belgium

## Slide 16
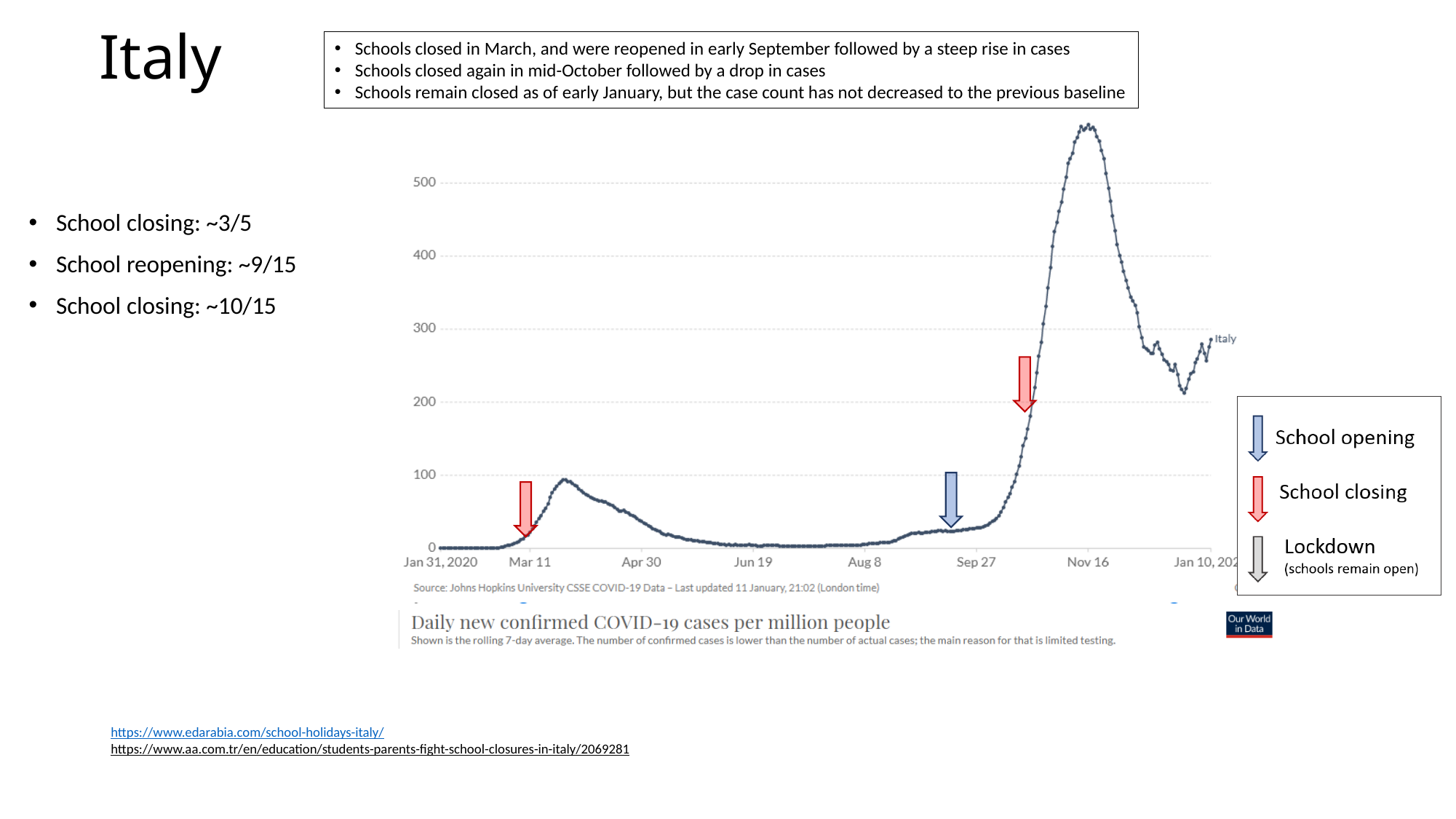

# Italy
Schools closed in March, and were reopened in early September followed by a steep rise in cases
Schools closed again in mid-October followed by a drop in cases
Schools remain closed as of early January, but the case count has not decreased to the previous baseline
School closing: ~3/5
School reopening: ~9/15
School closing: ~10/15
https://www.edarabia.com/school-holidays-italy/
https://www.aa.com.tr/en/education/students-parents-fight-school-closures-in-italy/2069281

## Slide 17
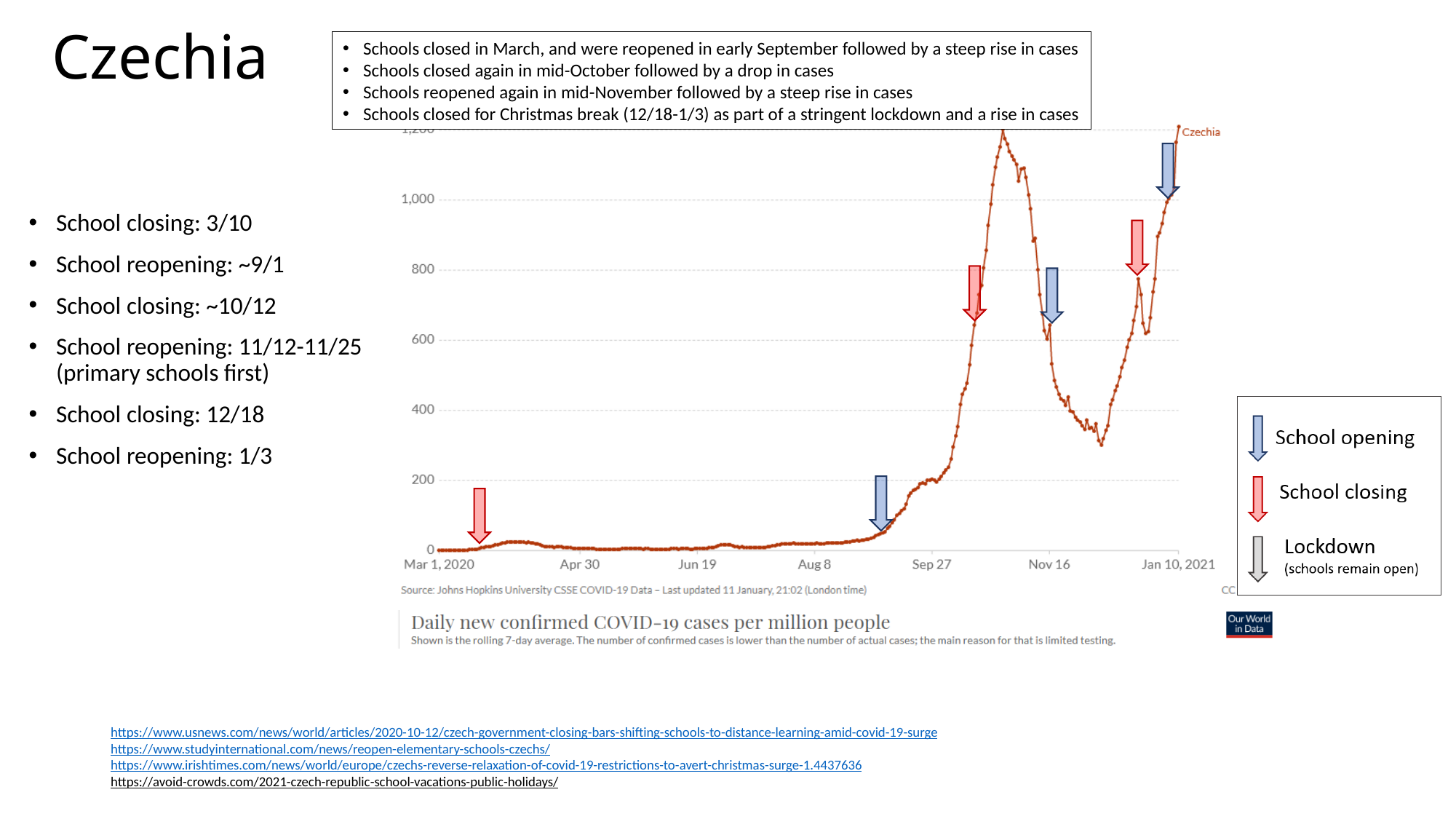

# Czechia
Schools closed in March, and were reopened in early September followed by a steep rise in cases
Schools closed again in mid-October followed by a drop in cases
Schools reopened again in mid-November followed by a steep rise in cases
Schools closed for Christmas break (12/18-1/3) as part of a stringent lockdown and a rise in cases
School closing: 3/10
School reopening: ~9/1
School closing: ~10/12
School reopening: 11/12-11/25 (primary schools first)
School closing: 12/18
School reopening: 1/3
https://www.usnews.com/news/world/articles/2020-10-12/czech-government-closing-bars-shifting-schools-to-distance-learning-amid-covid-19-surge
https://www.studyinternational.com/news/reopen-elementary-schools-czechs/
https://www.irishtimes.com/news/world/europe/czechs-reverse-relaxation-of-covid-19-restrictions-to-avert-christmas-surge-1.4437636
https://avoid-crowds.com/2021-czech-republic-school-vacations-public-holidays/

## Slide 18
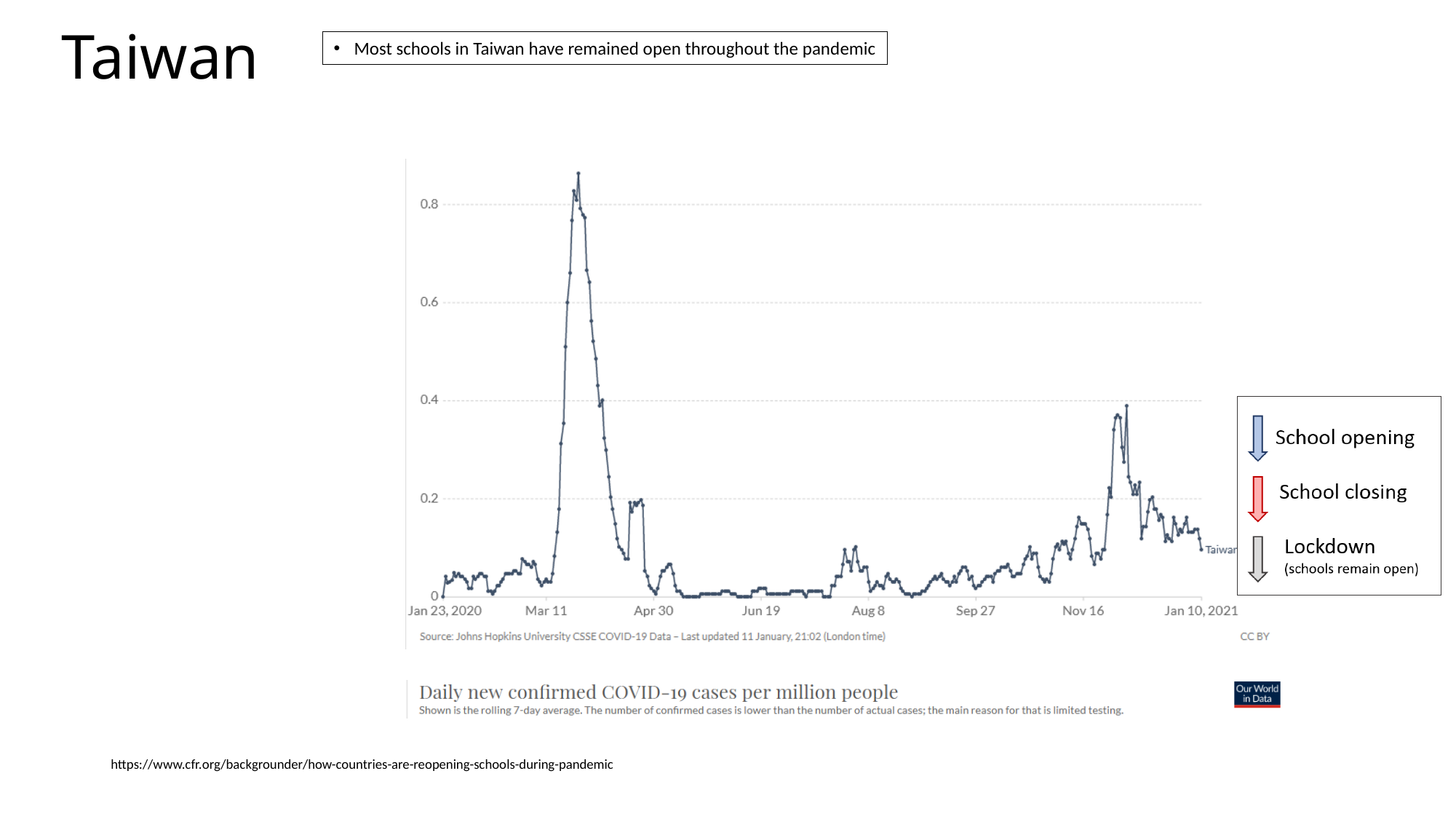

# Taiwan
Most schools in Taiwan have remained open throughout the pandemic
https://www.cfr.org/backgrounder/how-countries-are-reopening-schools-during-pandemic

## Slide 19
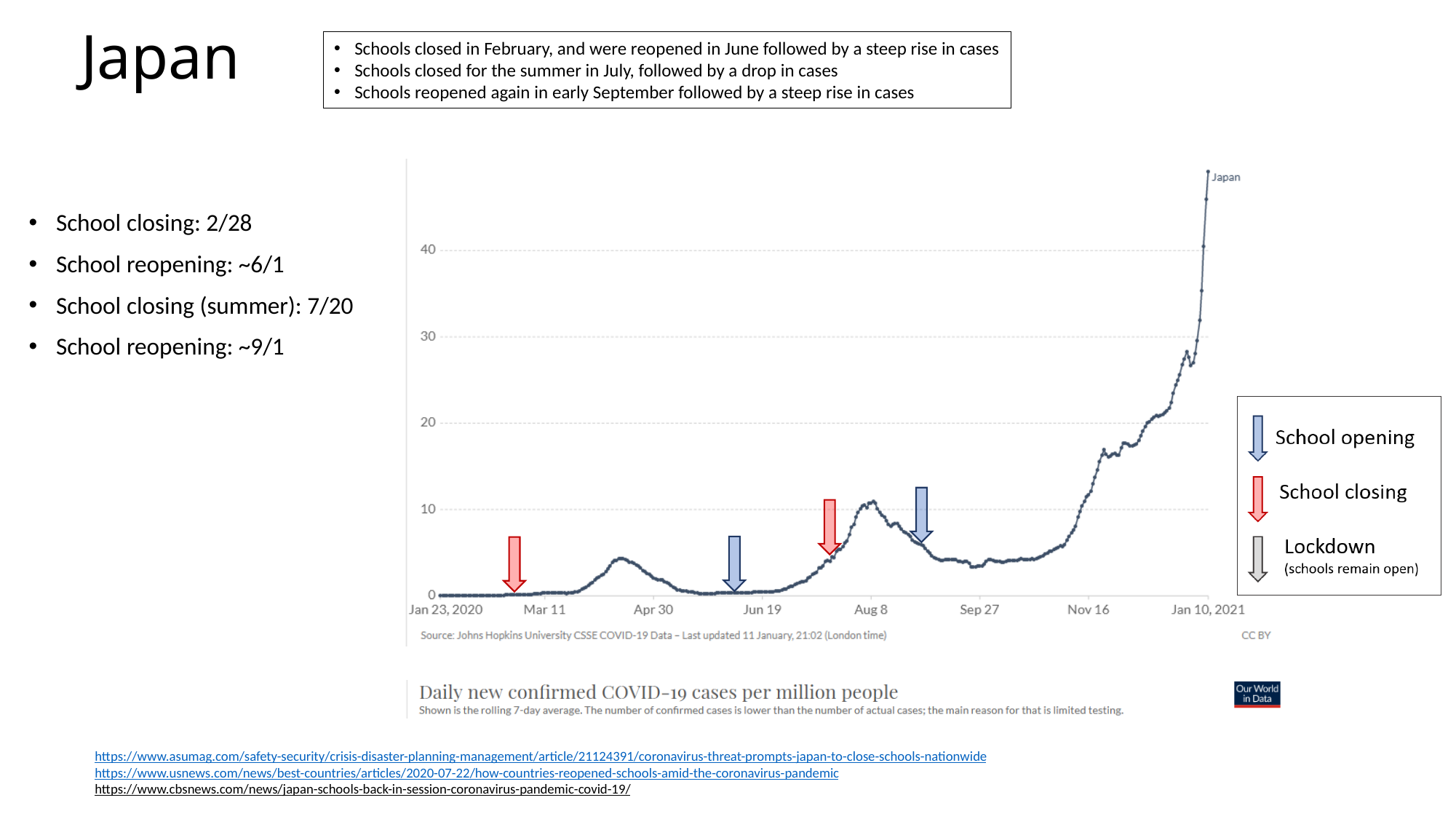

# Japan
Schools closed in February, and were reopened in June followed by a steep rise in cases
Schools closed for the summer in July, followed by a drop in cases
Schools reopened again in early September followed by a steep rise in cases
School closing: 2/28
School reopening: ~6/1
School closing (summer): 7/20
School reopening: ~9/1
https://www.asumag.com/safety-security/crisis-disaster-planning-management/article/21124391/coronavirus-threat-prompts-japan-to-close-schools-nationwide
https://www.usnews.com/news/best-countries/articles/2020-07-22/how-countries-reopened-schools-amid-the-coronavirus-pandemic
https://www.cbsnews.com/news/japan-schools-back-in-session-coronavirus-pandemic-covid-19/

## Slide 20
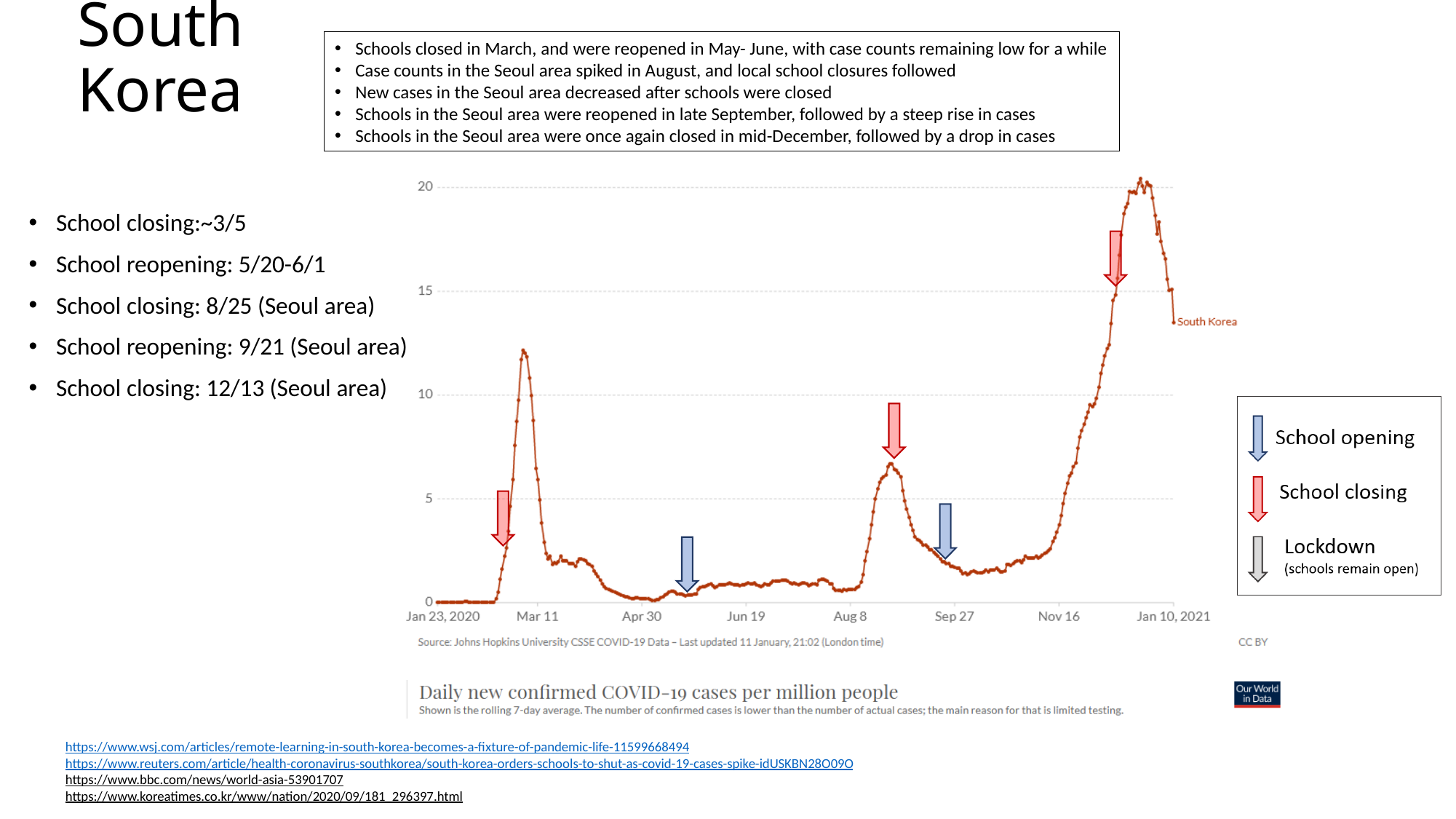

# South Korea
Schools closed in March, and were reopened in May- June, with case counts remaining low for a while
Case counts in the Seoul area spiked in August, and local school closures followed
New cases in the Seoul area decreased after schools were closed
Schools in the Seoul area were reopened in late September, followed by a steep rise in cases
Schools in the Seoul area were once again closed in mid-December, followed by a drop in cases
School closing:~3/5
School reopening: 5/20-6/1
School closing: 8/25 (Seoul area)
School reopening: 9/21 (Seoul area)
School closing: 12/13 (Seoul area)
https://www.wsj.com/articles/remote-learning-in-south-korea-becomes-a-fixture-of-pandemic-life-11599668494
https://www.reuters.com/article/health-coronavirus-southkorea/south-korea-orders-schools-to-shut-as-covid-19-cases-spike-idUSKBN28O09O
https://www.bbc.com/news/world-asia-53901707
https://www.koreatimes.co.kr/www/nation/2020/09/181_296397.html

## Slide 21
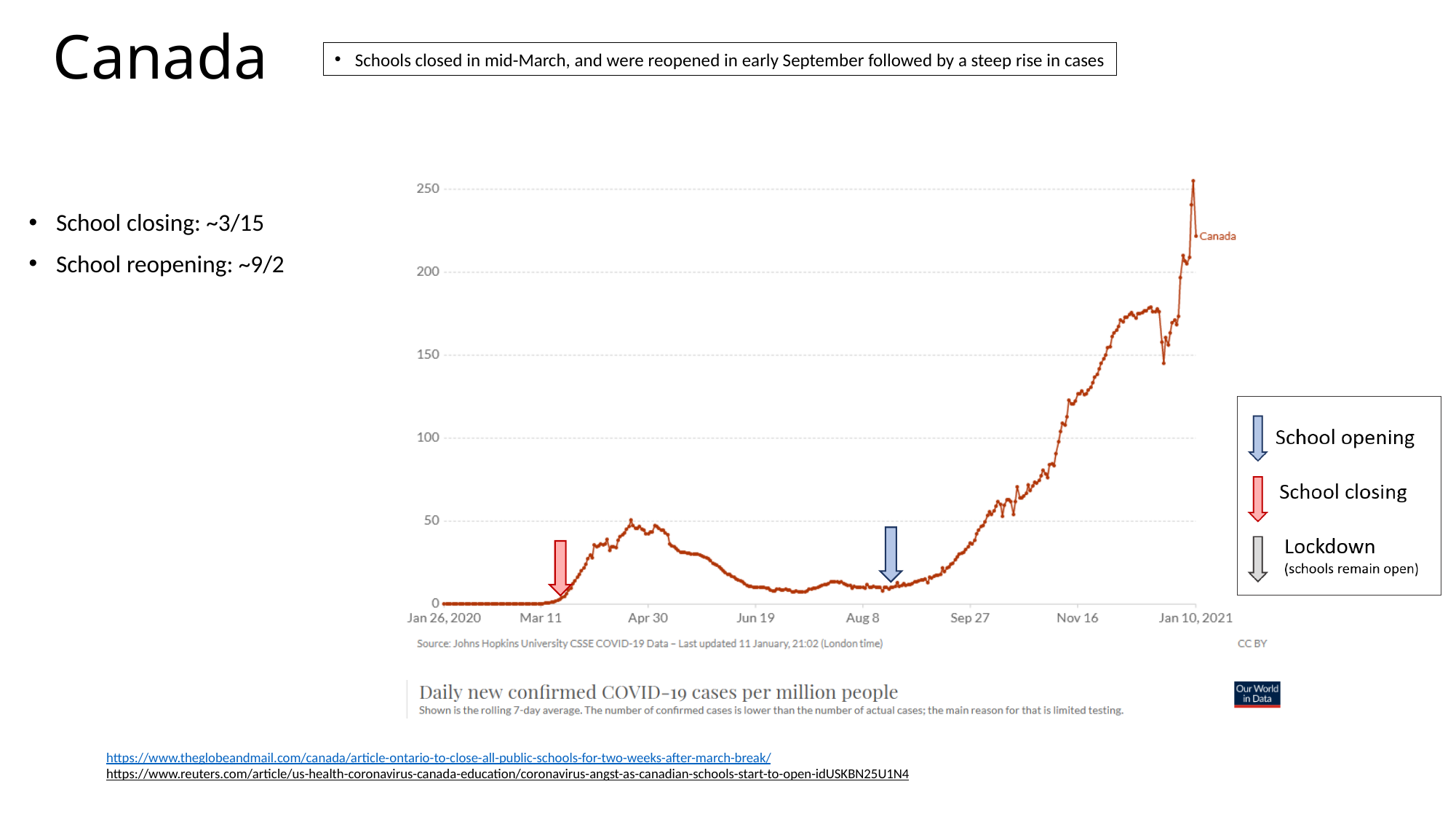

# Canada
Schools closed in mid-March, and were reopened in early September followed by a steep rise in cases
School closing: ~3/15
School reopening: ~9/2
https://www.theglobeandmail.com/canada/article-ontario-to-close-all-public-schools-for-two-weeks-after-march-break/
https://www.reuters.com/article/us-health-coronavirus-canada-education/coronavirus-angst-as-canadian-schools-start-to-open-idUSKBN25U1N4

## Slide 22
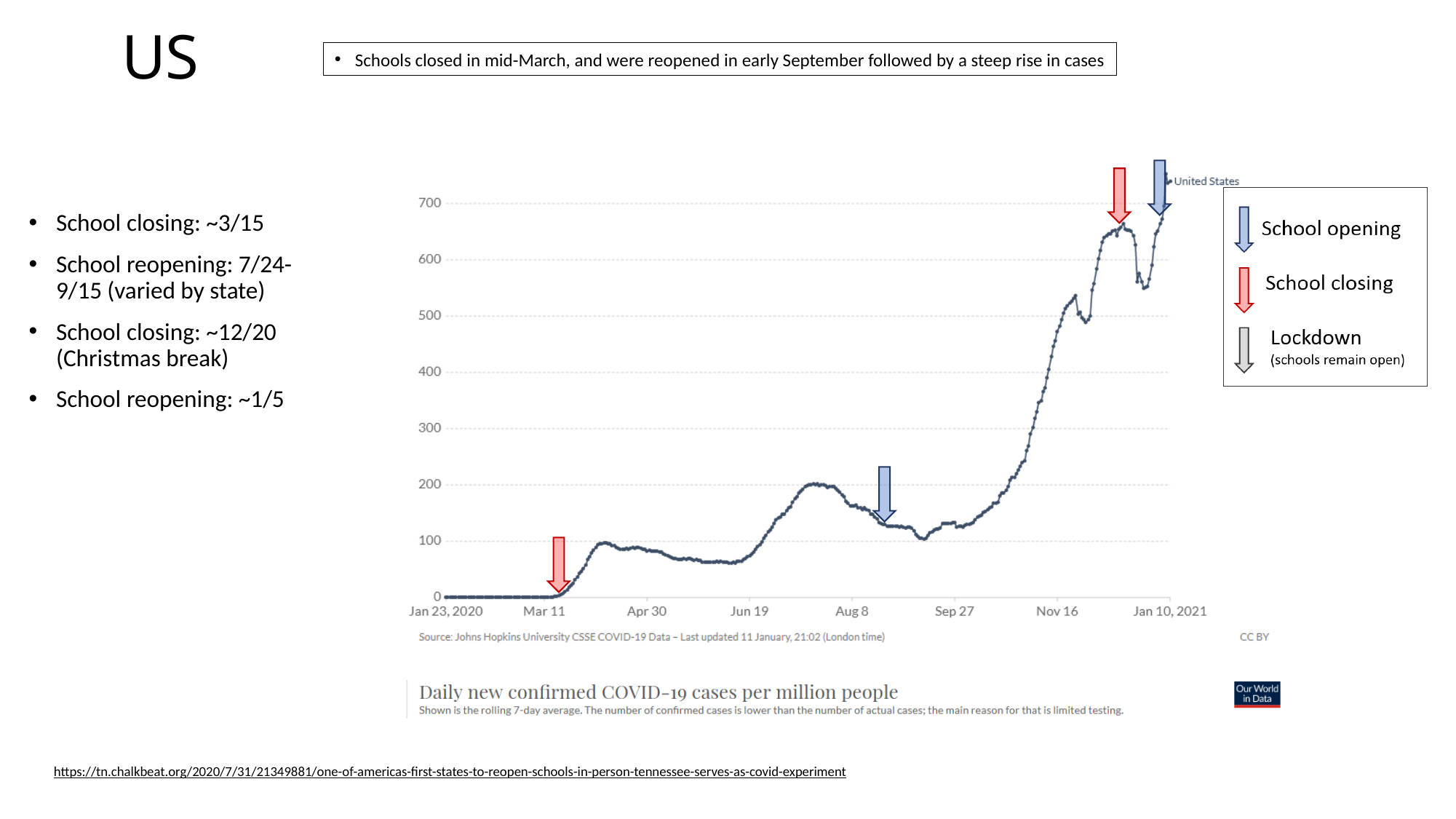

# US
Schools closed in mid-March, and were reopened in early September followed by a steep rise in cases
School closing: ~3/15
School reopening: 7/24-9/15 (varied by state)
School closing: ~12/20 (Christmas break)
School reopening: ~1/5
https://tn.chalkbeat.org/2020/7/31/21349881/one-of-americas-first-states-to-reopen-schools-in-person-tennessee-serves-as-covid-experiment
